# Superoxide Activates Ferroptosis via the Haber‐Weiss Reaction and Enhances Age‐Related Macular Degeneration

**DOI:** 10.1111/acel.70195

**Published:** 2025-08-10

**Authors:** Ying Huang, Zhenxing Zhou, Mengjia Huan, Qi Guo, Xiaoqian Zhang, Ruiqi Lu, Lushu Chen, Xiumiao Li, Jin Yao, Qin Jiang, Yong Xu

**Affiliations:** ^1^ The Affiliated Eye Hospital Nanjing Medical University Nanjing P. R. China; ^2^ Institute of Pediatric Children's Hospital of Soochow University Suzhou P. R. China; ^3^ The Second Affiliated Hospital Nanjing Medical University Nanjing P. R. China; ^4^ The Fourth School of Clinical Medicine Nanjing Medical University Nanjing P. R. China

**Keywords:** blue light, dry AMD, ferroptosis, GPX4, hydroxyl radical, MnSOD, superoxide

## Abstract

Antioxidant decline is crucial to driving age‐related macular degeneration (AMD). Ferroptosis, a regulated cell death mediated by iron‐dependent hydroxyl radical‐catalyzed phospholipid peroxidation through the Fenton reaction, is implicated in various chronic degenerative diseases. Here, we show that superoxide activates ferroptosis in retinal pigment epithelium (RPE) cells via the Haber‐Weiss reaction, thereby contributing to dry AMD. We silenced manganese superoxide dismutase (MnSOD/SOD2) in RPE cells and exposed the cells to blue light to induce ferroptosis by increasing superoxide anions. Additionally, MnSOD deficiency triggered the Hsp70‐linked ubiquitin‐dependent degradation of GPX4, further aggravating ferroptosis. We validated blue light‐induced ferroptosis in the RPE layer as a driver of the dry AMD phenotype in *Sod2* ^*+/−*^ mice. Consequently, SOD mimetics efficiently protected RPE against phototoxicity by reducing superoxide‐activated ferroptosis. Iron chelators or overexpressing GPX4 sufficiently eradicated ferroptosis. The finding reveals that excessive superoxide contributes to phospholipid peroxidation, providing a promising approach for preventing dry AMD by elevating MnSOD to inhibit RPE cell ferroptosis.

## Graphical abstract

Superoxide activates ferroptosis through the Haber‐Weiss reaction and contributes to the development of age‐related macular degeneration. MnSOD‐depleted RPE cells are susceptible to blue light‐induced ferroptosis. MnSOD deficiency promotes Hsp70‐driven ubiquitin‐mediated degradation of GPX4.

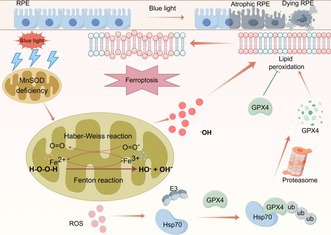

## 1 Introduction

Dry age‐related AMD is caused by accumulated small yellow deposits (drusen) between retinal RPE and Bruch's membrane, which can grow and lead to blurred vision (Ambati and Fowler 2012; Fleckenstein et al. 2021; Wong et al. 2014). RPE, located in the outermost layer of the retina, is a monolayer of cells that tightly forms a barrier and protects photoreceptor cells by maintaining retinal homeostasis. Thus, dysfunction of the RPE is crucial for the development of dry AMD, particularly oxidative stress‐mediated RPE injury, which becomes the core lesion in disease progression (Brown et al. 2019; Datta et al. 2017). During the aging of RPE cells, the ability of cells to scavenge reactive oxygen species (ROS) is reduced due to the decline in the function of the antioxidant defense system (Cai et al. 2000; Tokarz et al. 2013). ROS‐induced programmed cell death plays a pivotal role in the development of retinal degeneration diseases, including apoptosis and ferroptosis (Sun et al. 2024; Wenzel et al. 2005).

Ferroptosis, a regulated cell death characterized by iron‐dependent phospholipid peroxidation, results from hydroxyl radicals generated through the Fenton reaction, leading to cellular membrane structure instability (Jiang et al. 2021). Since mitochondria are the primary source of intracellular ROS generation and iron homeostasis, ferroptosis is relevant to mitochondrial function. Studies have shown that ferroptosis causes mitochondrial damage, characterized by alterations in membrane ultrastructure (Li et al. 2023). In addition to apoptosis, ferroptosis is involved in AMD (Sun et al. 2024). The ferrous ion content in the retinal tissues derived from dry AMD patients was higher than that of age‐matched normal retinal tissues, suggesting that iron metabolic disorders are associated with dry AMD (Hahn et al. 2003; Zhao et al. 2021). Inhibition of ferroptosis was more effective in rescuing RPE cells than inhibiting apoptosis or necrosis (Totsuka et al. 2019). Glutathione peroxidase 4 (GPX4) serves as a master surveillance hub to detoxify ferroptosis by catalyzing the conversion of lipid hydroperoxide to lipid alcohol (Yang et al. 2014). Activating glutathione (GSH) and inhibiting heme oxygenase‐1 (HO‐1) are essential to alleviating ferroptosis‐induced RPE cell death (Sun et al. 2018; Tang et al. 2021). In addition to GPX4, recent studies have discovered that glutathione‐independent inhibitors act as ferroptosis surveillance mechanisms. For instance, FSP1 diminishes lipid peroxidation by activating CoQ oxidoreductase, and sex hormone receptors‐regulated MBOAT1/2 inhibit ferroptosis by remodeling the phospholipid profile (Bersuker et al. 2019; Liang et al. 2023).

Superoxide anion, a precursor of ROS, is produced by complexes I and III in the mitochondrial electron transport chain by transferring an electron to an oxygen molecule. It is an active species involved in reversible chemical reactions and biological systems (Fridovich 1995). MnSOD (*SOD2*, or *Sod2* in murine), a primary antioxidant enzyme located in mitochondria, is critical to protect mitochondria by catalyzing superoxide into hydrogen peroxide that is further detoxified into water by peroxidases (Hoshida et al. 2002). In the Fenton reaction, hydrogen peroxide forms hydroxyl radical by catalyzing ferrous ions to ferric ions (Shen et al. 2018). Additionally, hydroxyl radicals can be produced by superoxide reacting with hydrogen peroxide by an iron closed‐loop catalytic cycle through the Haber–Weiss reaction (Koppenol 2022). Silencing *SOD2* in RPE cells facilitates the establishment of a dry AMD mouse model (Justilien et al. 2007), and the deprivation of *SOD2* in cancer cells enhances chemosensitivity via ROS‐induced ferroptosis (Su et al. 2023). However, the role of MnSOD and superoxide in ferroptosis remains poorly understood.

This study found that superoxide contributes to ferroptosis via the Haber‐Weiss reaction in RPE cells. MnSOD deficiency enhanced blue light–induced RPE cell injury by increasing lipid peroxidation and mitochondrial dysfunction. As a result, SOD mimetic and elevating GPX4 efficiently protected RPE cells from superoxide‐activated ferroptotic damage.

## 2 Results

### 2.1 MnSOD Decline Increases Blue Light‐Induced RPE Cell Death

To evaluate the relationship between antioxidant enzymes and dry AMD, we measured key antioxidant enzyme activities in the serum from age‐matched dry AMD patients and control donors, including Cu/Zn SOD, Mn SOD, GPX, and catalase. The results showed that Cu/Zn SOD and Mn SOD activities were lower in patients than in controls (Figure S1A–C). GPX activities were also reduced in patients compared to controls, although there were no significant differences in catalase activities between the two groups (Figure S1D,E). To explore whether lipid peroxidation is related to dry AMD, we quantified malondialdehyde (MDA) levels in the serum samples. As antioxidant levels declined, MDA levels were higher in patients than in controls, indicating that lipid peroxidation is involved in dry AMD (Figure S1F). The ages of patients and controls are listed in Table S1. Additionally, we measured enzyme activities and MDA content in retinal and choroidal tissues from established mice with dry AMD. The activities of SODs, GPX, and catalase decreased, while MDA content increased in mice with dry AMD compared to control mice (Figure S2A–F).

**FIGURE 1:**
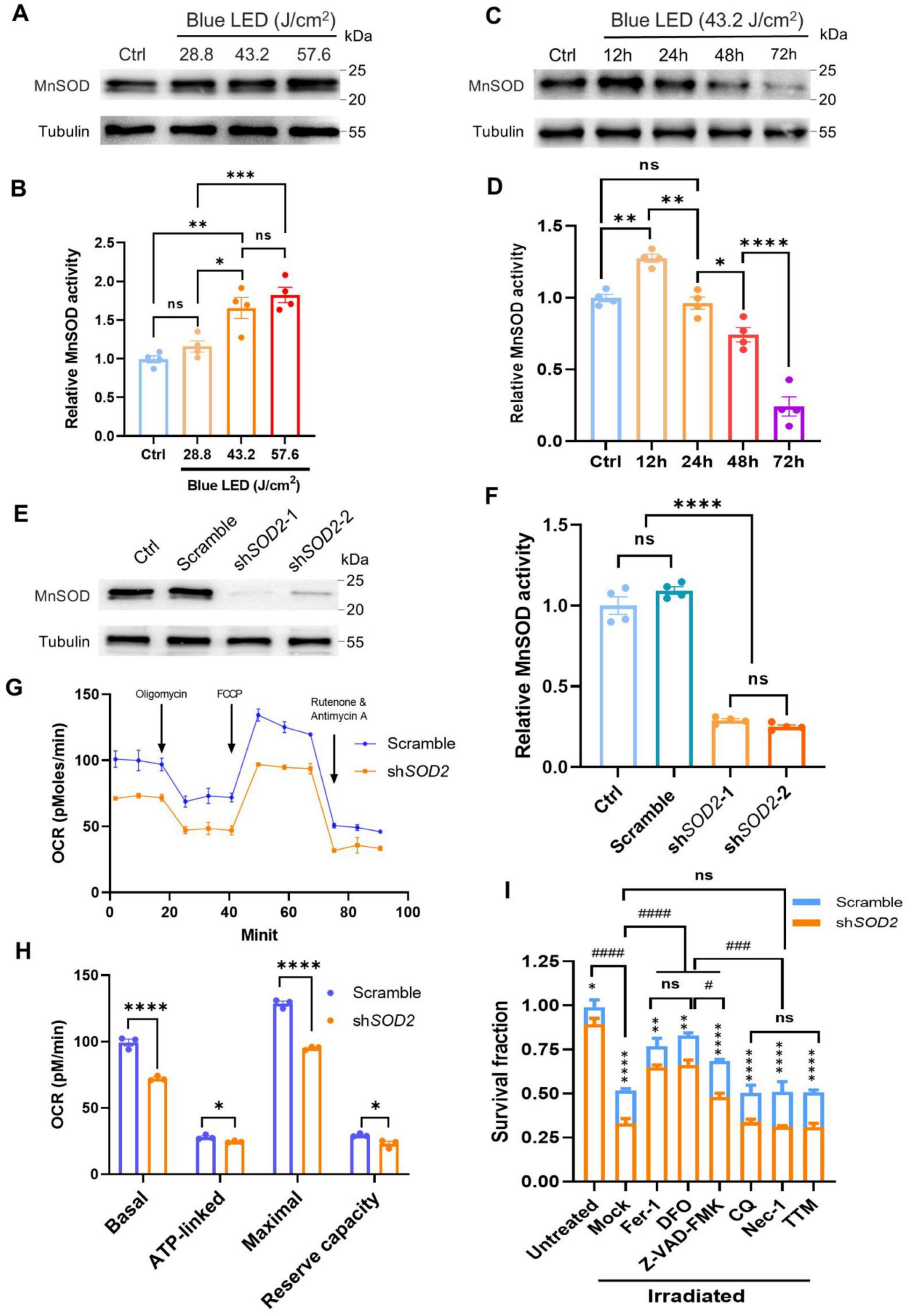
The effects of MnSOD silence on mitochondrial respiration and cell death. (A) Immunoblots of MnSOD in RPE cells after 12 h exposure to dose‐escalated blue light. (B) The relative MnSOD activities in the irradiated cells (*n* = 4). (C) Immunoblots of MnSOD in RPE cells after 12–72 h exposure to blue light. (D) The relative MnSOD activities in the irradiated cells at different time points (*n* = 4). (E) Immunoblots of MnSOD in MnSOD‐silenced RPE cells. (F) The reduction of MnSOD activities in MnSOD‐silenced RPE cells (*n* = 4). (G) Mitochondrial oxygen consumption rate (OCR) in the MnSOD‐deprived RPE cells. Oligomycin, FCCP, and antimycin/rotenone were automatically injected to measure OCR levels at basal, ATP‐linked, maximal, and background, respectively. (H) Quantification of OCR at different stages, the reserve capacity of respiration obtained by the maximal OCR subtracting the basal OCR (*n* = 3). (I) Quantification of cell survival rates using a CCK‐8 assay. Multiple inhibitors for inhibiting different types of programming cell death pathways were included and mock serves as their vehicle control. Fer‐1 and DFO for inhibiting ferroptosis, Z‐VAD‐FMK for inhibiting apoptosis, CQ for inhibiting autophagy, Nec‐1 for inhibiting necroptosis, and TTM for inhibiting cuproptosis (*n* = 3). The results are presented as the mean ± SD. *p < 0.05, **p < 0.01, ****p < 0.0001, ns, no significance (*p* > 0.05); ^#^ *p* < 0.05, ^###^ *p* < 0.001, ^####^ *p* < 0.0001 showing the comparisons in multiple groups (I); *t*‐test in (H), one‐way ANOVA in (B, D, F), two‐way ANOVA in (I).

To examine the role of MnSOD in protecting RPE cells, we increased oxidative damage by exposing the cells to a dose‐escalated blue light‐emitting diode (LED). Blue light raised MnSOD expression and enzymatic activity during the early adaptive response at 12 h (Figure 1A,B). However, the MnSOD response declined after 24 h due to accumulated phototoxicity (Figure 1C,D), which aligns with the decline observed in patients. Therefore, we silenced MnSOD in RPE cells to induce superoxide accumulation using a lentivirus carrying shRNA targeting the *SOD2* gene (Figure 1E,F). Consequently, MnSOD depletion led to reduced mitochondrial respiration (Figure 1G,H) and a slight decrease in cell proliferation (Figure S3A,B).

To examine the mechanisms underlying ROS‐induced RPE cell death, we pretreated the cells with multiple cell death inhibitors before blue light irradiation. The silence of MnSOD strikingly enhanced blue light‐induced cell death. Among inhibitors, ferroptosis inhibitors (Fer‐1 and DFO) and an apoptosis inhibitor (Z‐VAD‐FMK) protected cells from phototoxicity. In contrast, there were no significant protective effects from CQ, Nec‐1, and TTM, suggesting that autophagy, necroptosis, and cuproptosis were not associated with blue light‐induced cell death (Figure 1I). Notably, inhibition of ferroptosis was more efficient than inhibition of apoptosis in MnSOD‐silenced cells, indicating that ferroptosis may be a predominant form of oxidative stress‐induced cell death. These results suggest that MnSOD deficiency is critical for enhancing ROS‐induced RPE cell death, mainly through increasing ferroptosis.

### 2.2 MnSOD Deficiency Enhances Blue Light‐Induced ROS

**FIGURE 2:**
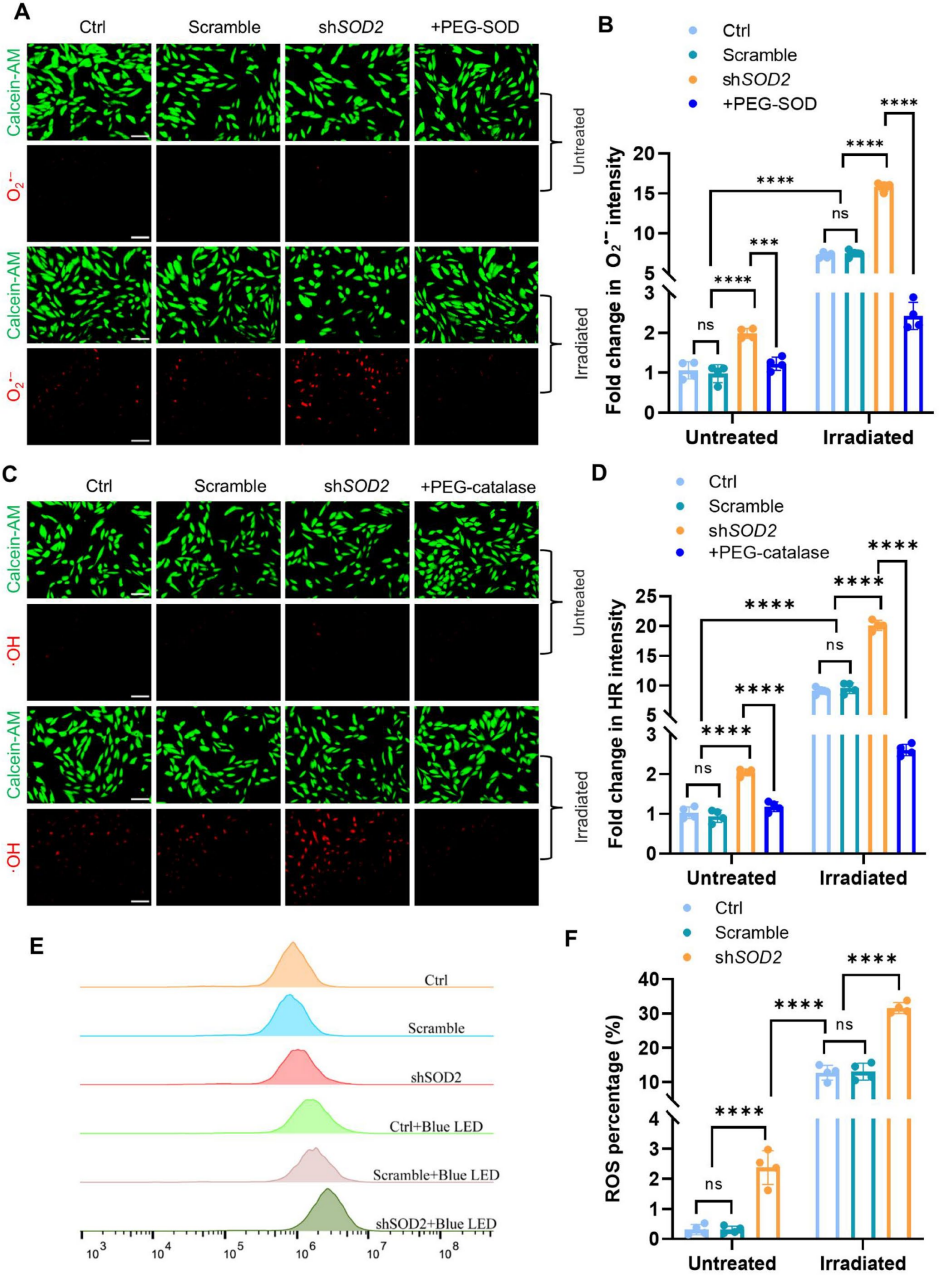
Quantification of superoxide anion and hydroxyl radical in irradiated RPE cells. (A) Representative image of superoxide anion in MnSOD‐deprived RPE cells. (B) Flow cytometry quantifying superoxide anion in the different groups (*n* = 4). (C) Representative image of hydroxyl radical (HR) in RPE cells. (D) Flow cytometry quantifying HR in different groups (*n* = 4). (E) Flow cytometry analyzing total ROS in RPE cells. (F) Quantification of total ROS in different groups (*n* = 4). The results are presented as the mean ± SD. ****p < 0.0001, ns, no significance (*p* > 0.05), two‐way ANOVA. The scale of bars: 100 μm (A, C).

To define the function of MnSOD in maintaining cellular redox homeostasis in RPE cells, we quantified the levels of superoxide anions in the cells using a DHE fluorescent probe. As shown in Figure 2A,B, blue light irradiation increased cellular superoxide compared to untreated control, and silencing MnSOD further accumulated superoxide that was removed by PEG‐SOD. In addition, we quantified hydroxyl radical levels in the cells using a specific probe. Consistently, blue light highly increased the cellular hydroxyl radical levels, and silencing MnSOD enhanced the irradiation effect that was eliminated by PEG‐catalase (Figure 2C,D). The increased cellular ROS levels were quantified by flow cytometry to measure the intensity of the FITC channel (Figure 2E,F). Furthermore, we quantified mitochondrial superoxide using a mitoSOX probe and mitochondrial hydroxyl radical by colocalizing the hydroxyl radical image with a mitotracker. Silencing MnSOD enhanced the mitochondrial superoxide and hydroxyl radical levels in irradiated cells (Figure S3C–E). In addition, we silenced MnSOD in mouse primary RPE cells (Figure S4A–C). Consistently, blue light increased the levels of superoxide anion and hydroxyl radical in the cells (Figure S4D–F).

We further examined apoptosis in irradiated cells. Compared to untreated controls, blue light irradiation resulted in an apoptotic rate of 4% in RPE cells and increased the rate to 12% in MnSOD‐silenced cells (Figure S5A,B). However, apoptotic cell death is much less than blue light‐induced total cell death, as shown in Figure 1I, indicating that apoptosis is not a prominent type of cell death in irradiated RPE cells. Consistent with the apoptotic cells detected, the ratio of Bcl2 to Bax decreased, and caspase 3 protein and activity increased in the irradiated cells (Figure S5C–E).

### 2.3 Superoxide Activates RPE Cell Ferroptosis and Leads to Mitochondrial Damage

**FIGURE 3:**
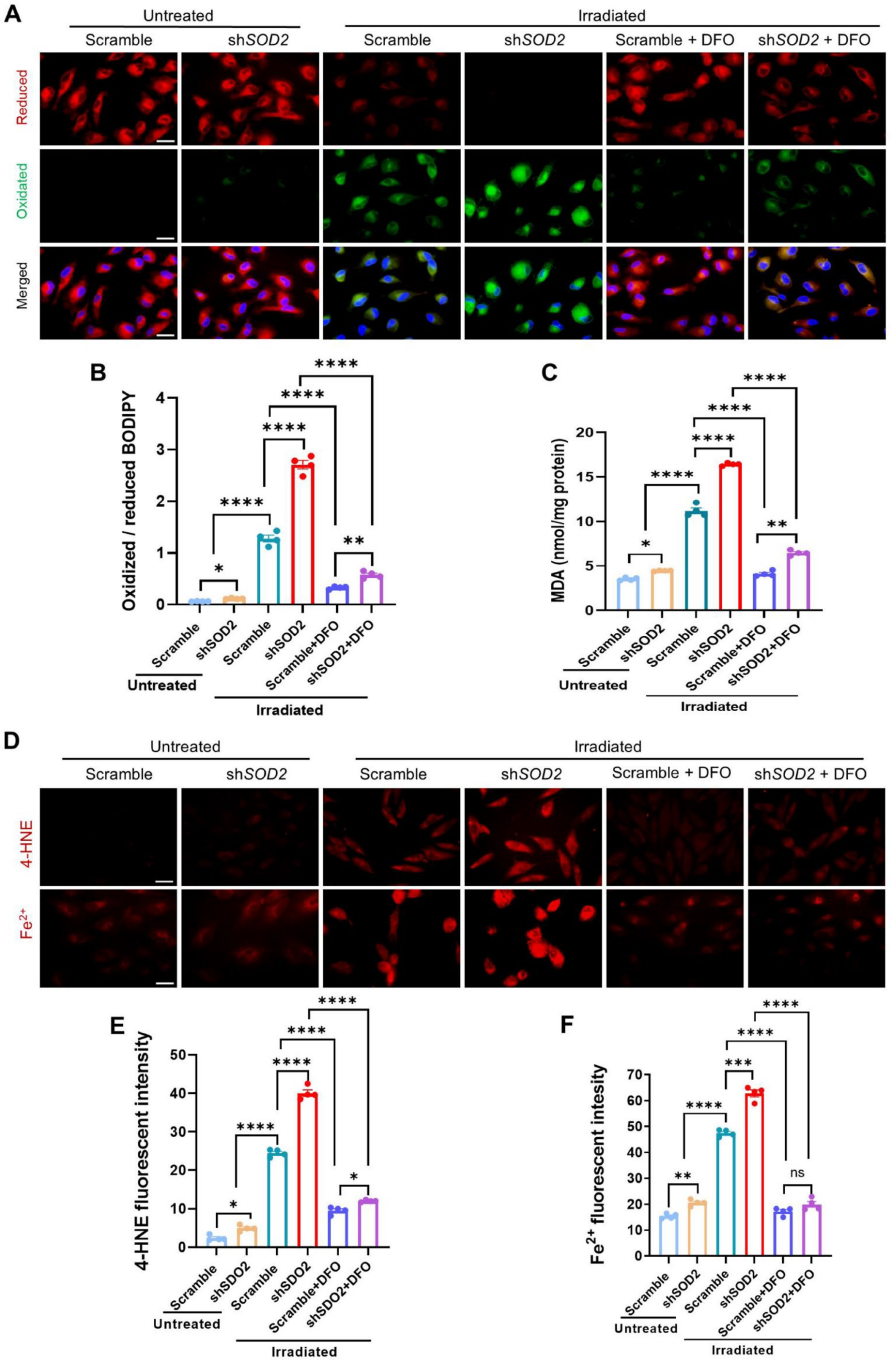
Quantification of lipid peroxidation, ferrous ion, and 4‐HNE in irradiated RPE cells. (A) Representative images of oxidized and reduced lipids in MnSOD‐deprived RPE cells exposed to blue light. (B) The ratio of oxidized probe to reduced probe in the cells (*n* = 4). (C) Quantification of the MDA level in the cells (*n* = 4). (D) Fluorescent images of 4‐HNE and ferrous ion in the cells. (E, F) Flow cytometry quantifying 4‐HNE and ferrous ions in the cells. The results are presented as the mean ± SD. *p < 0.05, **p < 0.01, ****p < 0.0001, two‐way ANOVA. The scale of bars: 40 μm (A, D).

To assess the effect of increased ROS on the induction of lipid peroxidation, we quantify oxidized and reduced lipids in irradiated cells using a BODIPY C11 probe. As shown in Figure 3A,B, blue light irradiation increased the oxidized lipid form but decreased the reduced lipid form compared to untreated controls. Silencing MnSOD further boosted the ratio of the oxidized lipids to the reduced lipids in the irradiated group, even though it had a slight effect on the untreated group. Notably, the ferroptosis inhibitor DFO was efficient in diminishing ROS‐induced lipid oxidation. Flow cytometry further quantified the levels of increased lipid oxidation (Figure S6A,B). Consistently, silencing MnSOD highly increased MDA levels in the irradiated cells, and DFO could eliminate the effect (Figure 3C). Since 4‐Hydroxy‐2‐nonenal (4‐HNE) serves as a cytotoxic lipid peroxidation marker (Liu et al. 2023), we confirmed that blue light irradiation increased 4‐HNE expression with the high content of ferric iron (Fe^2+^), and the effect was enhanced in MnSOD‐silenced cells (Figure 3D–F). Moreover, zonula occludens‐1 (ZO‐1) is crucial to maintaining RPE cell function by organizing tight junction components (Obert et al. 2017). Correspondingly, blue light irradiation reduced the ZO‐1 image, particularly in MnSOD‐silenced cells, indicating an essential role of MnSOD in maintaining RPE structure (Figure S6C,D). In addition, blue light also increased the levels of lipid peroxidation and cell survival in the primary RPE cells, particularly in MnSOD‐silenced cells. DFO efficiently protected the cells by reducing lipid peroxidation, confirming that blue light irradiation causes RPE cell death by inducing ferroptosis (Figure S7A–C).

### 2.4 Superoxide‐Activated Ferroptosis Exacerbates Blue Light‐Induced Retinal Injury

**FIGURE 4:**
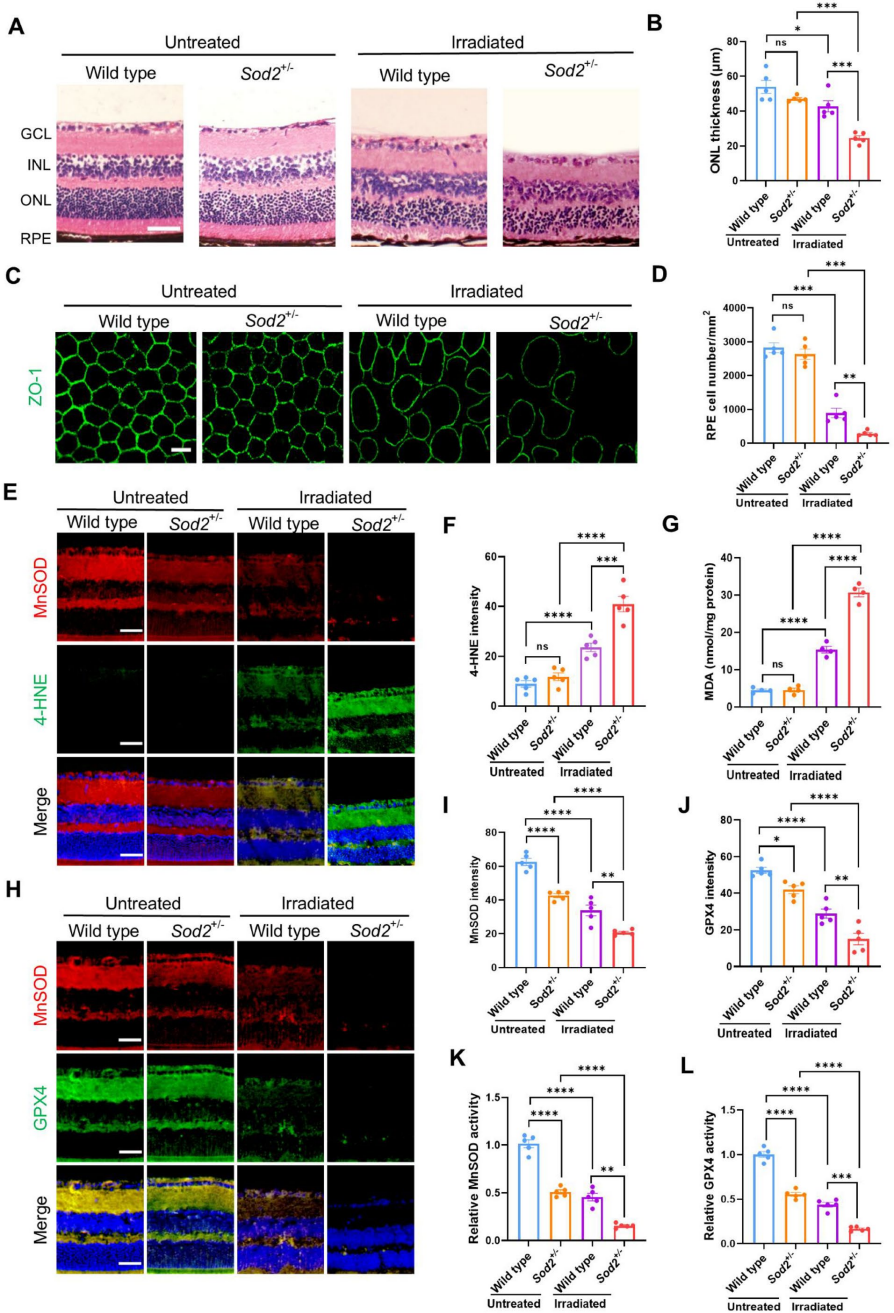
Ferroptotic damage in rental tissues of *Sod2* ^*+/−*^ mice exposed to blue light. (A) H&E pathological images of phototoxicity in mice retinal tissues. GCL, ganglion cell layer; INL, inner nuclear layer; ONL, outer nuclear layer, and RPE layer. (B) Quantification of the thickness of ONL layers in mice rental tissues (*n* = 5). (C) Fluorescent imaging RPE layer cell integrity using a ZO‐1 fluorescent antibody. (D) Quantification of RPE cell numbers in the tissues (*n* = 5). (E) Colocalization of 4‐HNE and MnSOD in the tissues with DAPI nuclear staining. (F, G) Quantification of the intensity of 4‐HNE and the content of MDA in the tissues (*n* = 5). (H) Colocalization of GPX4 and MnSOD with DAPI nuclear staining in the tissues. (I, J) Quantification of MnSOD and GPX4 images in the tissues (*n* = 5). (K, L) Quantification of MnSOD and GPX4 activities in the tissues (*n* = 5). The results are presented as the mean ± SD. *p < 0.05, **p < 0.01, ***p < 0.001, ****p < 0.0001, ns, no significance (*p* > 0.05), two‐way ANOVA. Scale bars: 50 μm in (A, E, H), 20 μm in (C).

To accumulate blue light‐induced superoxide, we further knocked out the *Sod2* gene in mice using the CRISPR/Cas9 gene editing system. Since homogeneous sod2^−/−^ genotype mice pups died from heart failure, we selected heterozygous *sod2* ^+/−^ *genotype* mice with normal physiological conditions for in vivo study. The genotype was confirmed to generate the protein frameshift due to four base pairs being deleted (Figure S8A). Accordingly, MnSOD expression level and enzymatic activity were reduced by 50% in *sod2* ^+/−^ mice (Figure S8B,C). Subsequently, we established a dry AMD model using *sod2* ^+/−^ mice exposed to blue light. Compared to untreated controls, irradiation changed the retinal anatomical structure with reduced thickness of the outer nuclear layer and increased RPE layer atrophy, particularly in *sod2* ^+/−^ mice (Figure 4A,B). MnSOD deficiency enhanced the effect of irradiation on the reduction of RPE layer cell junctions by increasing cell injury (Figure 4C,D).

We further imaged the tissue sides with fluorescence‐conjugated antibodies against MnSOD, GPX4, and 4‐HNE to verify whether ferroptosis is involved in blue light‐induced retinal damage. As expected, MnSOD deficiency increased 4‐HNE levels in the tissues from irradiated mice and boosted the MDA levels in the tissue extracts (Figure 4E–G, Figure S8D). Intriguingly, the combination of irradiation and MnSOD deficiency dramatically reduced GPX4 expression and activity (Figure 4H–L, Figure S8D–F), indicating that MnSOD deficiency downregulation of GPX4 expression is necessary for ferroptosis‐mediated RPE layer injury. These results suggested that MnSOD deficiency plays dichotomous roles in boosting ROS and suppressing GPX4.

### 2.5 MnSOD Deprivation Leads to Ubiquitin‐Mediated GPX4 Degradation

**FIGURE 5:**
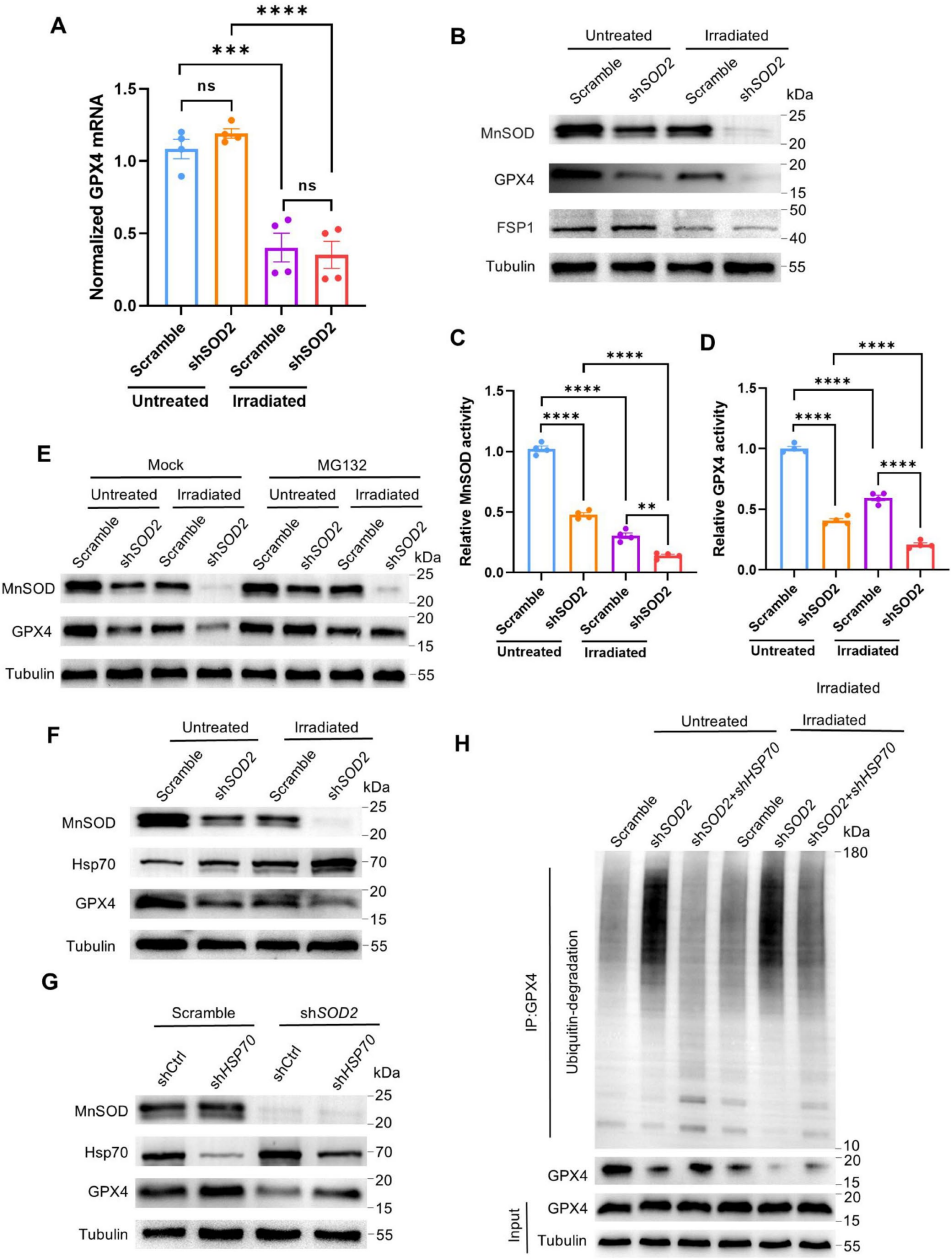
GPX4 ubiquitin‐degradation in MnSOD‐deprived RPE cells. (A) RT‐qPCR Quantifying GPX4 mRNA in RPE cells (*n* = 4). (B) Immunoblots of MnSOD and GPX4 in the cells. (C, D) Relative MnSOD and GPX4 activities in the cells (*n* = 4). (E), Immunoblots of GPX4 in the cells treated with MG132, and mock serves as its vehicle control. (F) Immunoblots of MnSOD, Hsp70, and GPX4 protein in the cells. (G) Immunoblots of MnSOD, GPX4, and Hsp70 in MnSOD‐Hsp70 double silenced RPE cells. (H) Immunoblotting analyses of GPX4 ubiquitin‐degradation in the cells. GPX4 antibody immunoprecipitated cellular extracts and then immunoblotted with a ubiquitin antibody to assess the effects of MnSOD and Hsp70 on the GPX4 ubiquitin‐degradation. The results are presented as the mean ± SD, *n* = 4. **p < 0.01, ***p < 0.001, ****p < 0.0001, ns, no significance (*p* > 0.05), two‐way ANOVA.

In addition to GPX4, recent studies demonstrated that FSP1 functions to inhibit ferroptosis through a GPX4‐independent pathway (Doll et al. 2019). Thus, we examined the expression of GPX4 and FSP1 in MnSOD‐silenced RPE cells. Although blue light exposure reduced GPX4 mRNA levels, MnSOD deprivation did not affect GPX4 mRNA expression (Figure 5A). However, MnSOD silence strikingly reduced GPX4 protein levels and relative activities in irradiated cells while slightly affecting FSP1 protein levels (Figure 5B–D). The results indicated that MnSOD deprivation may downregulate GPX4 expression at the posttranslational regulation level. Thus, we examined whether the ubiquitin‐proteasome pathway is involved in GPX4 degradation using an MG132 proteasome inhibitor. Expectedly, MG132 efficiently diminished the effect of MnSOD silence on GPX4 degradation (Figure 5E).

Heat shock protein 70 (Hsp70) is a critical chaperone in the ubiquitin‐proteasome system in response to oxidative stress to maintain protein homeostasis (Wu et al. 2021). In this regard, we found that MnSOD silence and irradiation synergically increased Hsp70 expression, which was correlated with GPX4 protein degradation (Figure 5F). Furthermore, we double‐silenced Hsp70 and MnSOD in RPE cells, and depriving Hsp70 was resistant to the effect of MnSOD depletion on GPX4 expression (Figure 5G). Notably, silencing Hsp70 efficiently mitigated GPX4 ubiquitin degradation in MnSOD‐deprived cells (Figure 5H), suggesting that Hsp70 plays a vital role in GPX4 ubiquitination in response to ROS.

### 2.6 SOD Mimetic Suppresses Superoxide‐Activated Ferroptosis

**FIGURE 6:**
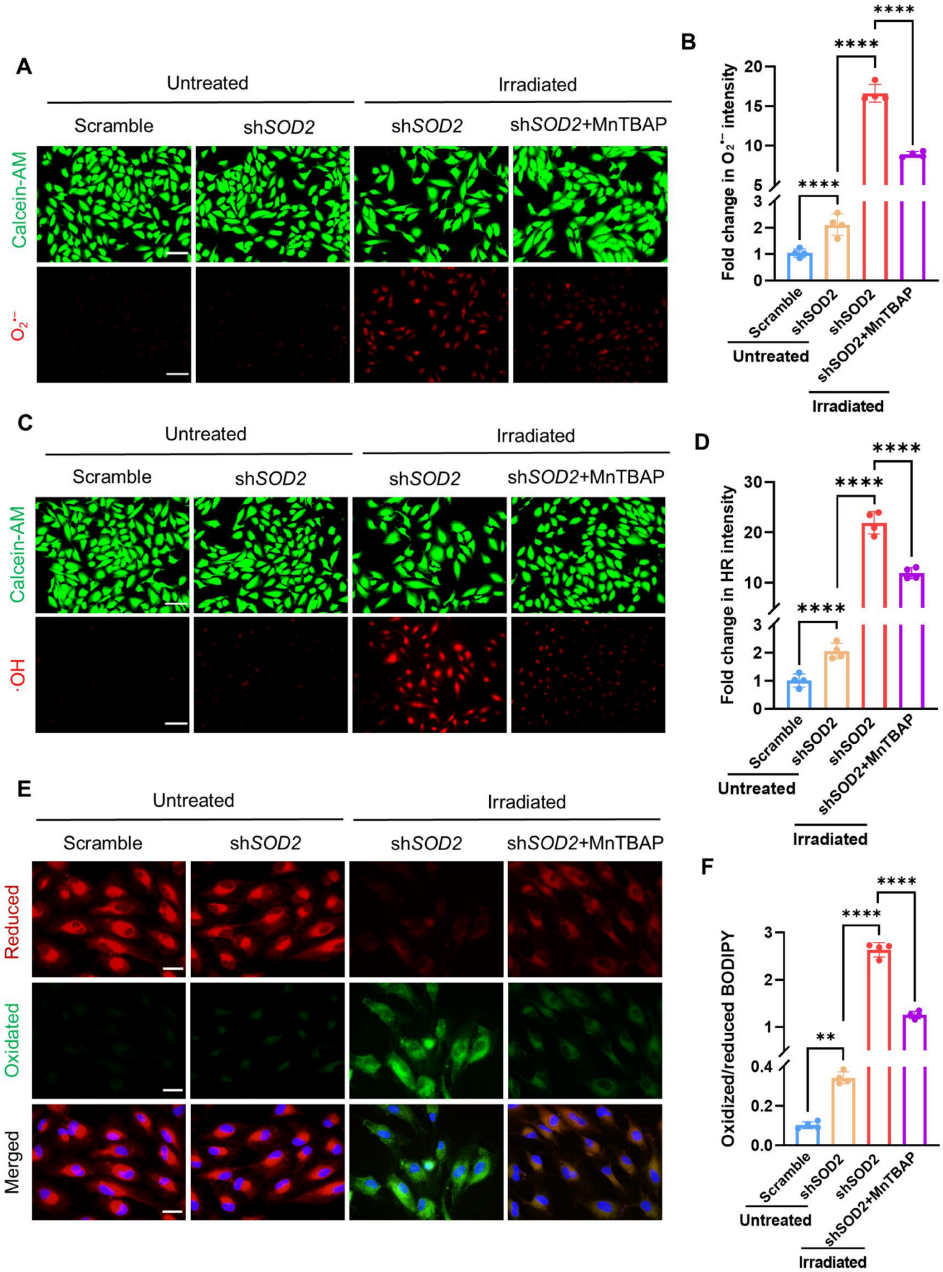
The effect of SOD mimetics on the protection of RPE cells against phototoxicity. (A) Fluorescent image of superoxide anion in RPE cells treated with SOD mimetic MnTBAP. (B) Flow cytometry quantifying superoxide anion in the cells (*n* = 4). (C) Fluorescent image of hydroxyl radical in the cells. (D) Flow cytometry quantifying hydroxyl radical in the cells (*n* = 4). (E) Fluorescent images of reduced and oxidized lipids in the cells. (F) Flow cytometry quantifying oxidized and reduced lipids to determine the ratio of oxidized lipids to reduced lipids in the cells (*n* = 4). The results are presented as the mean ± SD. **p < 0.01, ****p < 0.0001, two‐way ANOVA. Scale bars: 100 μm in (A, C), 40 μm in (E).

To verify superoxide‐induced RPE cell ferroptosis, we pretreated MnSOD‐silenced cells with a SOD mimetic, MnTBAP. The results showed that MnTBAP sufficiently removed superoxide anions and hydroxyl radicals induced by blue light irradiation in MnSOD‐silenced cells (Figure 6A–D). Correspondingly, MnTBAP dramatically reversed the ratio of oxidized lipids to reduced lipids by eliminating blue light‐induced lipid oxidation in the cells (Figure 6E,F). MnTBAP eliminated 4‐HNE, ferrous ion, and MDA levels in MnSOD‐silenced cells (Figure S9A–D) and consistently protected the cells against phototoxicity (Figure S9E). SOD mimetic functional compensation of MnSOD deficiency in antioxidant response confirmed that dysfunction of MnSOD plays a causal role in blue light‐induced RPE cell ferroptotic injury.

### 2.7 Overexpressing GPX4 Blocks Superoxide‐Activated Ferroptosis

**FIGURE 7:**
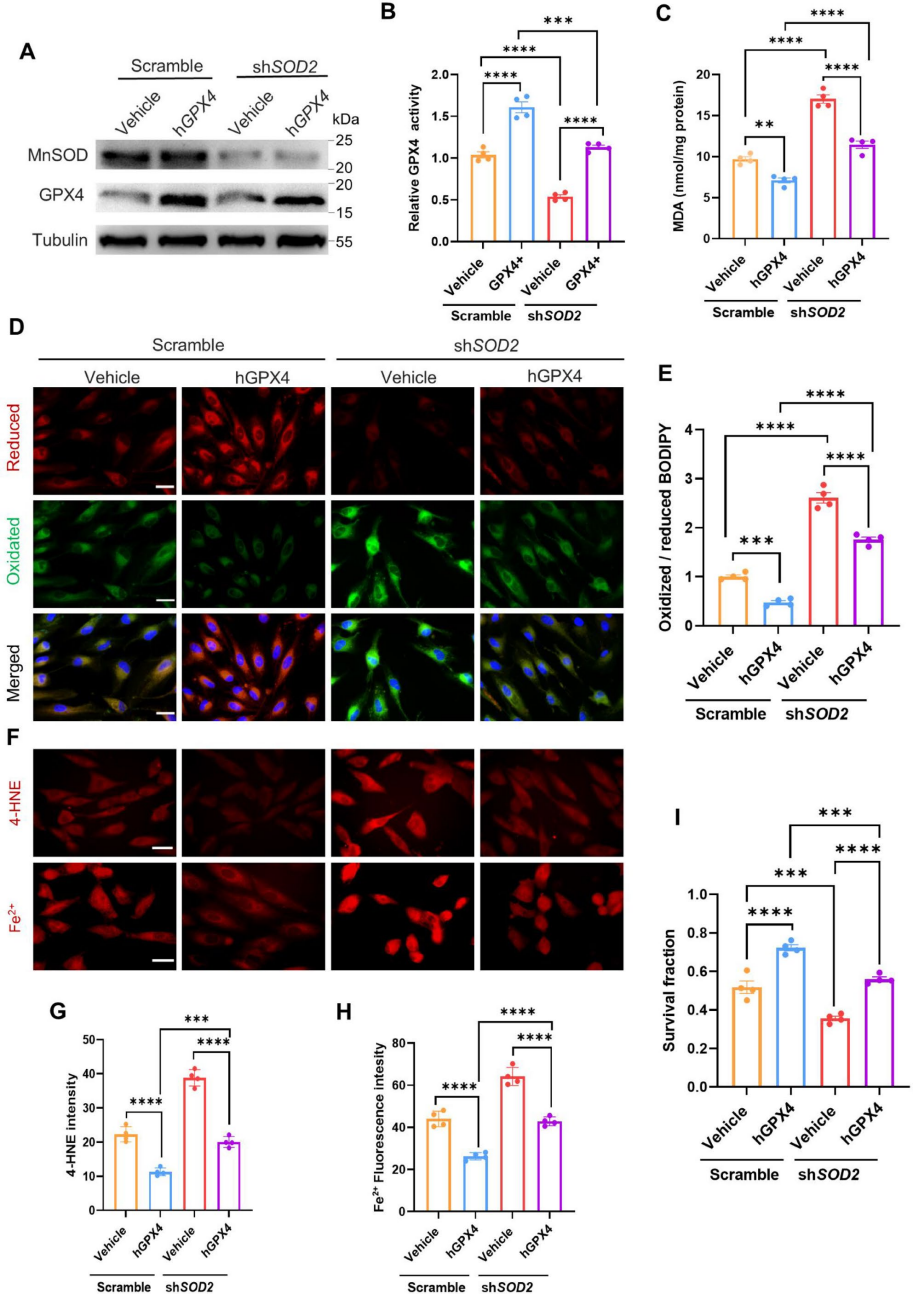
Suppression of RPE cell ferroptosis by overexpressing GPX4. (A) Immunoblots of MnSOD and GPX4 in GPX4‐overexpressed RPE cells. (B) The relative GPX4 activities in the cells (*n* = 4). (C) After exposure to blue light, the MDA levels in the cells (*n* = 4). (D) After exposure to blue light, fluorescent image of oxidized and reduced lipids in the cells. (E) The ratio of oxidized lipids to reduced lipids in the cells (*n* = 4). (F) Fluorescent image of 4‐HNE and ferrous ions in the cells. (G, H) Flow cytometry quantifying 4‐HNE and ferrous ions (*n* = 4). (I) GPX4 protective effect of RPE cell survival against phototoxicity (*n* = 4). The results are presented as the mean ± SD. **p < 0.01, ***p < 0.001, ****p < 0.0001, two‐way ANOVA. Scale bars: 40 μm (D, F).

To verify GPX4 protecting RPE cells from blue light‐induced ferroptosis, we ectopically expressed GPX4 in MnSOD‐silenced cells (Figure 7A,B). The rise of GPX4 efficiently reduced the blue light‐increased MDA levels in MnSOD‐silenced cells (Figure 7C). Correspondingly, the high level of GPX4 reduced the ratios of oxidized lipid to reduced lipid and GSH to GSSG (Figure 7D,E; Figure S10A–E). In addition, expressing GPX4 decreased 4‐HNE and ferrous ion levels (Figure 7F–H), thereby protecting cell survival against phototoxicity (Figure 7I). The results confirmed the MnSOD deprivation‐enhanced RPE cell ferroptosis, at least in part, through the inactivation of GPX4.

**FIGURE 8:**
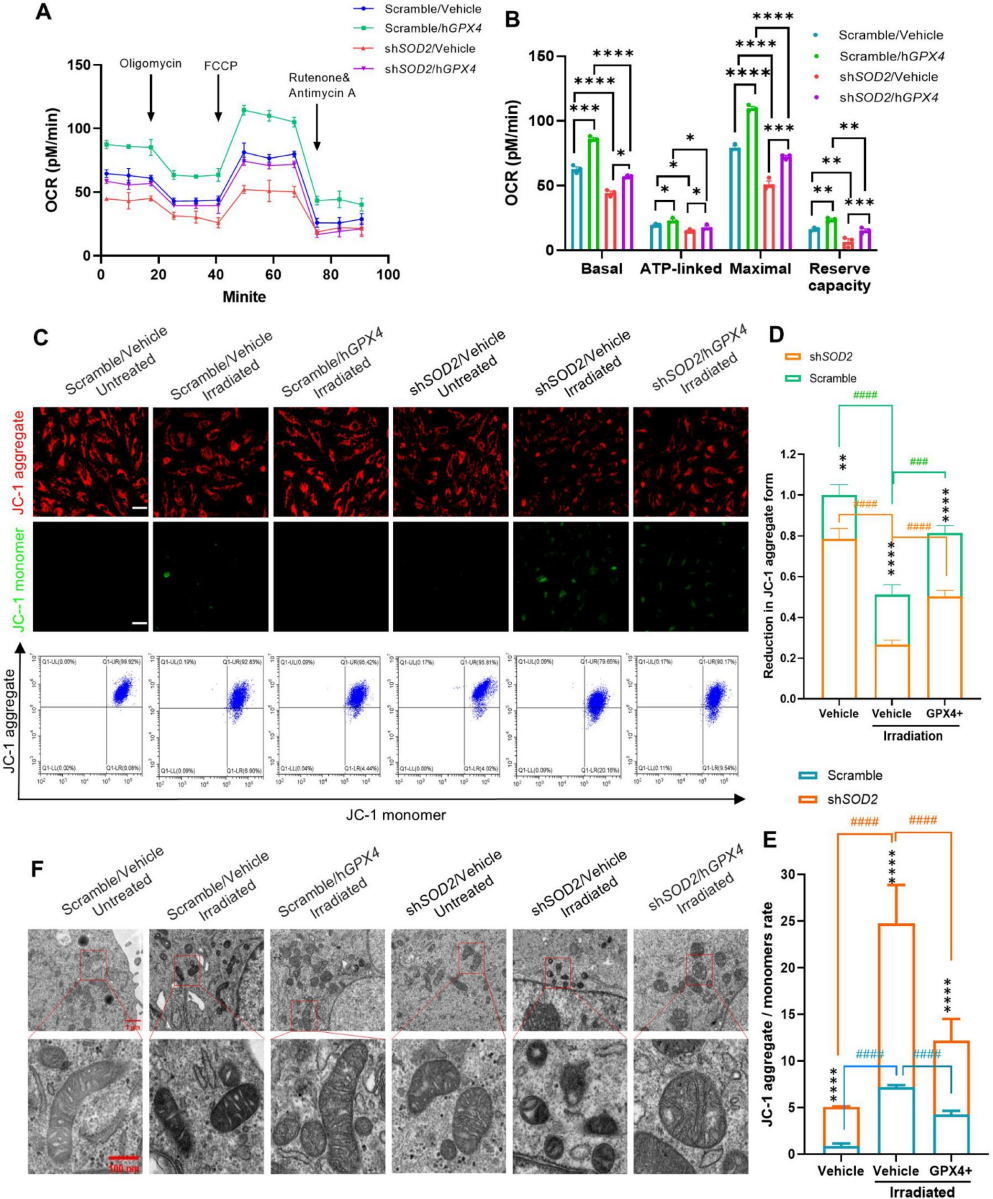
The effect of GPX4 on RPE cell mitochondrial function maintenance. (A) OCR analysis of GPX4‐overexpressed cells exposed to blue light. (B) Quantification of OCR at four different stages (*n* = 3). (C) Fluorescent images of JC‐1 aggregate form (red fluorescence) and monomer form (green fluorescence). Flow cytometry quantifying the JC‐1 aggregate and monomer forms. (D), Quantitative JC‐1 aggregate fluorescent intensity (*n* = 3). (E), Flow cytometry quantifying the JC‐1 monomer form (*n* = 3). (F) EM photograph of mitochondrial structures in the cells. The results are presented as the mean ± SD. **p < 0.01****p < 0.0001, comparisons in same groups. ^####^ *p* < 0.0001, comparisons in different groups, two‐way ANOVA. Scale bars: 40 μm in (C); 1 μm in (F) top panels, and 100 nm in (F) low panel.

To examine whether overexpressing GPX4 ameliorates mitochondrial function by eliminating ROS‐induced ferroptosis, we measured OCR in blue light‐irradiated RPE cells. Silencing MnSOD decreased mitochondrial OCR, but overexpressing GPX4 efficiently recovered mitochondrial respiration (Figure 8A,B). Blue light reduced the mitochondrial potential by decreasing the JC1 aggregate forms in the cells. Overexpressing GPX4 improved the mitochondrial potential by increasing the JC1 aggregate form and decreasing the JC1 aggregate form (Figure 8C–E). Blue light‐induced mitochondrial injury showed smaller mitochondrial size with decreased cristae, particularly in MnSOD‐silenced cells. However, elevating GPX4 was essential to recapture mitochondrial structure (Figure 8F). Overall, the insights into mitochondrial damage suggested that MnSOD deficiency is a crucial contributor to RPE cell injury by enhancing ROS‐induced ferroptosis, as illustrated in the Graphic Abstract.

## 3 Discussion

Age‐related AMD, a leading cause of irreversible blindness, is a daunting challenge in public health due to increasing morbidity among people older than 50 (Guymer and Campbell 2023). The mechanisms underlying AMD remain elusive, and effective intervention to control disease progression is urgently needed (Kumbhar et al. 2024). Overexposure to solar UV radiation and visible light is a high risk for AMD (Crooke et al. 2017; Cupini et al. 2023). Particularly, long‐term exposure to electrical device screens and LED lamps causes blurred vision, leading to a growing concern regarding blue light irradiation enhancing ocular degeneration in public health (Kwon et al. 2024; Ouyang et al. 2020). Blue light exposure produces excessive ROS and triggers oxidative damage in the retina (Kim et al. 2023). Monolayer RPE cells in the outermost layer of the retina are more vulnerable to phototoxicity (Marie et al. 2018). In addition to ROS‐induced apoptosis (Seagle et al. 2006), emerging evidence demonstrated that ferroptosis is a distinctive type of regulated cell death involved in RPE cell injury (Gupta et al. 2023; Li et al. 2024). Thus, the declining antioxidant defense system is widely recognized for promoting RPE cell death and AMD (Sun et al. 2018). This study demonstrated that ferroptosis is a dominant form of blue light‐triggered RPE cell death. The insight into mitochondrial damage suggested ferroptosis‐induced intracellular membrane lipid peroxidation rather than apoptosis‐induced DNA fragmentation.

A recent transcriptomic study has shown that RPE aging plays a crucial role in the development of dry AMD. Increased ROS and inflammation, along with decreased antioxidants such as GPX4, are related to lipid peroxidation in aged RPE (Dubey et al. 2025). Glutathione‐activated GPX4 is a central ferroptosis surveillance mechanism that detoxifies hydroxyl radical‐catalyzed iron‐dependent lipid peroxidation (Chen et al. 2021). In addition to GPX4, a previous study demonstrated that antioxidants such as SOD are upregulated in the early and intermediate stages of dry AMD patients, indicating an adaptive response that upregulates antioxidants to counteract increased ROS (Decanini et al. 2007). Subsequently, SOD deficiency contributes to RPE cell damage and retinal photoreceptor dysfunction (Brown et al. 2019). Superoxide reacts with hydrogen peroxide to produce hydroxyl radicals, catalyzed by cycling ferrous and ferric ions; i.e., the Haber–Weiss reaction (or cycle) was demonstrated in 1932 (Haber and Weiss 1932). The reaction was concluded as: first reaction, Fe^3+^ + O_2_^•−^ → Fe^2+^ + O_2_ second reaction, Fe^2+^ + H_2_O_2_ → Fe^3+^ + OH^−^ + ^**•**^OH; and net reaction, O_2_^•−^ + H_2_O_2_ → ^**•**^OH + OH^−^ + O_2_. However, the concept was later argued by criticizing the let reaction as a slow, thermodynamically unlikely process in soluble liquids (Koppenol 2001). Since the criticism was irrelevant to the biological system, the reaction was further supported by widely postulating that superoxide is a normal cellular metabolite and may produce hydroxyl radicals in vivo, although the basic reaction is slow in aqueous solution (Kehrer 2000). Even though the concept is still controversial in free radical biology (Koppenol 2022), this study clearly showed that MnSOD deficiency enhanced blue light‐induced ferroptosis by boosting the levels of superoxide and hydroxyl radical in RPE cells, supporting the concept revival in the biological system. Although the cytotoxicity of ferroptosis is mediated by hydroxyl radical via the Fenton reaction, the cycle of ferrous and ferric ions during the Haber–Weiss reaction is crucial for iron homeostasis to sustain ferroptosis.

The dysfunction of superoxide dismutase is closely associated with aging‐related neuron degeneration diseases (Bhaskaran et al. 2023). As the first antioxidant defense line, MnSOD in mitochondria functions to remove superoxide, a precursor of ROS (Guo et al. 2023). MnSOD deficiency enhances mitochondrial oxidative stress, leading to metabolic dysfunction in RPE and photoreceptor cells (Brown et al. 2019; Mao et al. 2014). A polymorphism (V16A) of the *SOD2* gene was associated with exudative AMD (Kimura et al. 2000). ROS‐induced apoptotic cell death was increased in RPE cells derived from *sod2* ^+/−^ mice (Kasahara et al. 2005). This study demonstrated that the levels of superoxide and hydroxyl radicals highly increased in blue light‐irradiated MnSOD‐deprived RPE cells, resulting in enhanced ferroptosis rather than other types of programmed cell death. Ferroptosis marker, lipid peroxidation level, mitochondrial damage, and relative RPE layer injury were consistently increased in irradiated *sod2* ^+/−^ mice. SOD mimetics efficiently protected the RPE cells against ferroptosis by reducing ROS. The results indicate that excessive mitochondrial ROS may induce mitochondrial membrane lipid peroxidation.

Decline of antioxidant defense is crucial for aging, and inactivation of GPX4 plays a fundamental role in ferroptosis. Thus, inhibition of GPX4 and depletion of its substrate glutathione are critical to trigger ferroptosis (Yang et al. 2014; Yong et al. 2024). Multiple mechanisms are involved in ferroptosis. Activation of p53 and NF2 sensitizes tumor cells to ferroptosis (Jiang et al. 2015; Wu et al. 2019). Inactivation of mTORC1 and Wnt/β‐catenin enhances tumor chemotherapy by activating ferroptosis (Wang et al. 2022; Zhang et al. 2021). Mitochondrial protein FUNDC2 promotes doxorubicin‐induced cardiomyopathy by activating ferroptosis (Ta et al. 2022). Targeting HO‐1‐mediated RPE ferroptosis protects against retinal degradation, including AMD (Chen et al. 2023; Tang et al. 2021). We recently demonstrated that RelB enhances breast cancer tamoxifen resistance by upregulating GPX4 to inhibit ferroptosis (Xu et al. 2023). Studies by other groups reported that E3 ligase‐linked ubiquitin degradation of GPX4 is a critical target in cancer treatment (Ding et al. 2021; Wang et al. 2023).

Heat shock proteins (Hsps) conserve the function of maintaining protein homeostasis through post‐translational modifications (Chiosis et al. 2023; Hu et al. 2022). Timosaponin AIII sensitizes lung cancer to ferroptosis by triggering Hsp90‐linked GPX4 ubiquitination (Zhou et al. 2023). ROS modulates Hsp70 function on proteostasis in response to the redox signaling in aging‐related diseases (Havalová et al. 2021; Zhang et al. 2022). We reported that exosome‐delivered Hsp70 enhances breast cancer adriamycin resistance by switching mitochondrial oxidative phosphorylation to glycolysis (Hu et al. 2021). Hsp70 serves as a crucial molecular chaperone for regulating E3 ligase‐triggered protein ubiquitin‐degradation in cancer and Alzheimer's diseases (Munari et al. 2022; Wu et al. 2021). This study demonstrated that Hsp70 implicates ubiquitin‐degradation of GPX4 in response to ROS, leading to lipid peroxidation and mitochondrial dysfunction.

In summary, this study unveiled that MnSOD‐deprived RPE cells are susceptible to blue light‐induced ferroptosis. Excessive superoxide‐enhanced ferroptosis suggests that the Haber–Weiss reaction is implicated in iron‐dependent lipid peroxidation. SOD mimetics efficiently protected RPE cells against ferroptosis by reducing superoxide and hydroxyl radicals. In addition, MnSOD deficiency motivated Hsp70‐linked ubiquitin degradation of GPX4 expression, leading to the attenuation of ferroptosis suppression. Insight into the depolarization of the mitochondrial membrane and impaired mitochondrial respiration predicts that the mitochondrial membrane may be a predominant target of superoxide‐induced ferroptosis.

## 4 Methods

### 4.1 Patients

Nanjing Medical University Eye Hospital collected peripheral blood samples from 15 patients newly diagnosed with dry AMD before treatment. To control the disease, blood samples from 20 age‐matched control donors were obtained from the Physical Examination Center, Nanjing Hospital, affiliated with Nanjing Medical University. The Ethics Committee of Nanjing Medical University approved the study protocol (protocol No. 2021011) and obtained written informed consent from the patients enrolled in this study.

### 4.2 Mice

According to the ARVO statement on animal use in ophthalmology, the Animal Care and Use Committee of Nanjing Medical University approved the mouse visual research protocol (No. IACUC‐2407047). The Animal Experimental Center of Jiangsu Huachuang Xinnuo Pharmaceutical Technology Co Ltd provided 8‐week‐old C57BL/6J male mice for this study. We knocked out mouse MnSOD using a CRISPR/Cas9 gene editing system targeting the *Sod2* gene. Since the homozygous gene type of *Sod2* ^*−/−*^ died after birth due to heart failure, we established the mouse dry AMD experimental model using the heterozygous gene type of *Sod2* ^*+/−*^ mice. The mice were placed in a dark room for 24 h, and their pupils were dilated by treating eyes with 1% cyclopentolate hydrochloride for 30 min. The mice were exposed to a blue LED light source of 1100 lx for 7 days in a cycle of 12 h irradiation and 12 h dark. After mice euthanasia, the excised eyeballs were placed in 4% paraformaldehyde for 2 h. Next, the anterior segment of the eye was removed and placed in 30% sucrose solution overnight for dehydration. The tissues were soaked in an optimal cutting temperature medium (Thermo Fisher Scientific) and stored at −80°C.

### 4.3 Tissue Image

After rinsing with 1 **×** PBS, tissues were soaked in a permeabilization solution (5% BSA + 1% Triton) for 1 h. The tissues were embedded in paraffin to section 5 μm tissue slides and then stained with hematoxylin–eosin (H&E) reagent (C0105S, Beyotime), and the pathological structures were photographed under a light microscope. For protein imaging, the tissues were incubated with primary rabbit antibodies against MnSOD (1:1000, ab13533, Abcam), GPX4 (1:1000, #59735, Cell Signaling Technology), and 4‐HNE (1:1000, ab46545, Abcam) overnight at 4°C. For imaging RPE cell sheeting, the neuroretina layer was carefully peeled off using a tweezer to avoid touching the RPE cell layer and incubated with a ZO‐1 antibody (1:1000, ab216880, Abcam) overnight at 4°C. After washing with 1 **×** PBS, the tissues were incubated with a fluorophore‐conjugated secondary antibody (1:1000, ab172329, Abcam) for 2 h in the dark. The tissues were counterstained with DPAI to localize nuclei. The images were captured using a fluorescence microscope (Olympus Corporation).

### 4.4 Cell Culture and Gene Manipulation

Human RPE cell line (ARPE‐19) and human lens epithelium (HLE) were obtained from the American Type Culture Collection (ATCC, USA). Murine primary RPE cell line was purchased from Xiamen Immocell Biotechnology, China. The RPE cells were cultured in DMEM/F12 medium (12400–024, Gibco) supplemented with 10% fetal bovine serum (FBS500‐S, AusGeneX, Australia) and 100 U/mL penicillin with 100 μg/mL streptomycin (15140148, Gibco) in a humidified atmosphere of 5% CO_2_ at 37°C. The primary RPE cells were characterized using a specific marker, RPE65, with a negative control, HLE (human lens epithelium) marker. The human *SOD2 and Hsp70* genes were silenced in RPE cells by infecting the cells with lentivirus‐coated shRNAs. Targeting sequences: *SOD2, 5′‐TTCTCCGAACGTGTCACGT*‐3′ and *Hsp70, 5′‐GGAAGGACGAGTTTGAGTTAT*‐3′, which were cloned into a GV112 lentiviral vector. Additionally, GPX4 was ectopically expressed in MnSOD‐deprived cells by infecting cells with a lentivirus carrying GPX4 cDNA in a GV208 lentiviral vector. The stable cell clones were selected using gentamicin (15750078, Invitrogen) or puromycin (A1113803, Invitrogen).

### 4.5 Cell Treatment

Blue light‐emitting diode (LED) was used as an irradiation source to treat RPE cells. The cells were plated in 6‐well or 96‐well plates at 70% cell density and cultured for 12 h. After washing with 1 **×** PBS, the cells were irradiated with energy‐escalated blue LED (28.8–57.8 J/cm^2^). After irradiation, the cells were cultured for 12–72 h to quantify ROS and lipid peroxidation. To remove blue light‐induced ROS, the cells were pretreated with 10 μM SOD mimetic MnTBAP (ab141496, Abcam) for 30 min.

### 4.6 ROS Quantification

After blue light exposure, the cells were incubated with 5 μM dihydroethidium (DHE) probe (ab23606, Abcam) and 1 μM mitoSOX fluorescent probe (M36005, Invitrogen) at 37°C for 30 min to analyze superoxide anion in RPE cells and mitochondria at 535/610 nm. PEG‐SOD (S9549, Sigma) was included as a positive control to remove superoxide. In addition, the cells were incubated with a 1000 **×** diluted hydroxyl radical detection fluorescent probe (ab219931, Abcam) at 37°C for 30 min to quantify hydroxyl radical at 535/675 nm, and PEG‐catalase (C4963, Sigma) was used to remove hydroxyl radical. The image was colocalized with a 200 nM mitotracker (HY‐135056, MCE) image to analyze mitochondrial hydroxyl radicals. To quantify total plating cells, the cells were labeled with a 2000 **×** diluted Calcein‐AM dye (65‐0853‐78, Invitrogen). The fluorescent images were analyzed using a fluorescence microscope (Nikon, Japan), and the fluorescent intensity was quantified using an M200 multifunction microplate reader. The relative superoxide and hydroxyl radical levels were calculated by normalizing the fluorescent probe‐detected images with the plating cell image. Additionally, total ROS levels were quantified by flow cytometry (BD Biosciences, USA) using a 1000 **×** diluted DCFH‐DA fluorescent probe (ab113851, Abcam).

### 4.7 Antioxidant Enzymatic Activity Assays

Cu/ZnSOD and MnSOD activities were measured using a SOD1 assay kit (E‐BC‐K022‐S, Elabscience) and a SOD2 assay kit (HCS232, Millipore). GPX and catalase activities were quantified using a GPX assay kit (353919, Millipore) and a catalase assay kit (E‐BC‐K031‐S, Elabscience). The enzymatic activities were normalized with the protein concentration. The GPX4 activity was measured using a specific chemical reaction described previously (Stolwijk et al. 2020). Briefly, the cells were homogenized in lysis buffer containing 100 mM Tris/Base (pH 8.0), 2.0 mM EDTA, 1.5 mM NaN3, 0.1% Triton X‐100, and 1 mM PMSF. The cell lysates were incubated with a reaction buffer (100 mM Tris/Base [pH 8.0], 2 mM EDTA, 1.5 mM NaN_3_, 0.1% Triton X‐100) containing glutathione reductase (1.5 U/mL), glutathione (3 mM), and NADPH (0.2 mM) for 5 min, and the enzymatic activity was measured at 340 nm by every 20 s for a total 5 min using an M200 multifunction microplate reader. During the first 100 s, the nonspecific rate of oxidation of NADPH was recorded. The GPX4‐specific enzymatic reaction was initiated by adding 30 μM phosphatidylcholine hydroperoxide substrate (P3782, Sigma), and then the GPX4‐dependent oxidation of NADPH was monitored for the next 200 s. The GPX4 activity was calculated as the rate of NADPH loss over time using an equation: [GPX4 activity] (mU/mg) = (Slope_PCOOH_—Slope_Background_) × [total reaction volume] (L) × 10^9^/[ε340 × d × protein (mg)] = ΔSlope × 57.996/mg protein, and then normalized to the cellular protein concentration. The cellular GSH and GSSG contents were measured using a GSH/GSSG assay kit (E‐BC‐K097‐M, Elabscience) according to the manufacturer's instructions. The ratio of GSH/GSSG was calculated by normalizing with protein concentration.

### 4.8 Cell Proliferation Assay

The cell proliferation was analyzed using a BeyoClick EdU kit (C0071S, Beyotime). Briefly, 10^4^ cells were plated in each well of the 24‐well plates overnight. The media containing 20 μM EdU was replaced and incubated for 3 h. After washing with 1× PBS, EdU‐positive cells, counterstained with DAPI, were observed under a fluorescence microscope.

### 4.9 Cell Survival Assay

After irradiation, the cells were plated in 96‐well plates at 2000 cells/well for 24 h. The cell survival rate was analyzed using a Cell Counting Kit‐8 (HY‐K0301, MCE) and measured with a microplate reader at 450 nm. The cell survival rate was normalized to the cell plating efficiency. For examining the potential programmed cell death mechanisms involved in irradiation‐provoked cell death, the cells were pretreated with multiple cell death inhibitors based on our previous investigation (Xu et al. 2023), including 50 μM deferoxamine mesylate (DFO, ab120727, Abcam), 10 mM ferrostatin‐1 (Fer‐1, ab146169, Abcam), 20 mM necrostatin‐1 (Nec‐1, ab141053, Abcam), 5 μM N‐acetylcysteine (NAC) adjusted to pH of 7.4 (ab284541, Abcam), 50 μM Z‐VAD (OMe)‐FMK (ab120487, Abcam), 50 μM chloroquine (CQ, ab142116, Abcam), and 20 μM tetrathiomolybdate (TTM, HY‐128530, MCE). Additionally, the cells were treated with 10 μM MnTBAP (ab141496, Abcam) to rescue cell survival by recovering the SOD activity. After 30 min of pretreatment, the cells were irradiated with blue light and then continuously cultured for 48 h. The cell survival fraction was measured using a CCK‐8 kit (MCE, JPY24500).

### 4.10 Quantification of Intracellular Ferrous Ions

The irradiated cells were incubated with 5 μM FeRhoNox‐1, a Fe^2+^ detection fluorescent probe (HY‐D1533, MCE) at 37°C for 1 h. After washing with 1 × PBS three times, the intracellular ferrous ion contents were imaged using an LSM 710 confocal microscope (Zeiss) at 532/570 nm.

### 4.11 Lipid Peroxidation Quantification

The lipid peroxidation levels were quantified using a BODIPY‐C11581/591 C11 fluorescent probe (10257152, Invitrogen) as described previously (Xu et al. 2023). In brief, the irradiated cells were incubated with 1 μM BODIPY probe and Hoechst at 37°C for 30 min. After washing with 1 **×** PBS, the cells were placed in glass‐bottom dishes, and the fluorescent density was quantified using confocal microscopy (Zeiss). The oxidized form (Green fluorescence) was acquired at 488/510 nm, and the reduced form (red fluorescence) was acquired at 581/591 nm. The cellular lipid peroxidation level was estimated using the ratio of the oxidized form to the reduced form. In addition, the level of lipid peroxidation was quantified using a FACSAira II SORP flow cytometer (BD) with the BODIPY probe.

### 4.12 Apoptosis Analysis

After irradiation, the cells were incubated with an Annexin‐V/PI/PI staining kit (abs50001‐25T, Vazyme) in the dark for 15 min. The apoptotic cell rate was analyzed by flow cytometry and normalized to the total number of planting cells.

### 4.13 Mitochondrial Respiration

After irradiation, the oxygen consumption rate (OCR) in living cells was measured by Seahorse XF Analyzer (Agilent Technologies). Briefly, the cells were seeded at a density of 10^5^ cells/well in Seahorse 96‐well plates and cultured in a DMEM/F12 complete medium at 37°C for 12 h in a 5% CO_2_ incubator. The OCR was measured under the following four conditions: no injection (basal), auto injection with 1 mM oligomycin, 1 mM FCCP, and 0.5 mM rotenone/antimycin. Each data point takes multiple reading values to calculate the average OCR rates.

### 4.14 Mitochondrial Structure

Irradiation‐mediated alteration of mitochondrial morphology was analyzed using a transmission electron microscope (TEM). The irradiated cells were embedded and then sliced to 100 nm using an RMC ultramicrotome and stained with uranyl acetate and lead citrate. The mitochondrial structure was examined using a Tecnai Spirit TEM (FEI) at an acceleration voltage of 120 kV.

### 4.15 Mitochondrial Membrane Potential

Mitochondrial membrane depolarization was monitored by JC‐1 staining. The irradiated cells were incubated with a 1:200 diluted JC‐1 fluorescent probe (T3168, Invitrogen) at 37°C for 20 min. After washing with 1× PBS twice, the cells were imaged using fluorescence microscopy (Olympus Corporation) and quantified by flow cytometry. The JC‐1 aggregated form (red fluorescence) and monomer form (green fluorescence) were acquired at 590 nm and 529 nm. The mitochondrial potential ΔΨm was assessed using a ratio of aggregated form to monomer form.

### 4.16 RT‐qPCR

Total RNA was extracted from cells using a Trizol Reagent (15596026, Invitrogen) and converted into cDNAs using the SuperScript IV First‐Strand Synthesis System (18091050, Invitrogen). cDNAs were quantified by qPCR with gene‐specific primers using a PowerUp SYBR Green Master Mix (A25742, Invitrogen). The mRNA levels were quantified by normalizing to the internal control tubulin signal. The primer sequences for GPX4: forward primer, 5′‐GAGGCAAGACCGAAGTAAACTAC‐3′, reverse primer, 5′‐CCGAACTGGTTACACGGGAA‐3′; for Tubulin: forward primer, 5’‐TGGACTCTGTTCGCTCAGGT‐3′, reverse primer, 5’‐TGCCTCCTTCCGTACCACAT‐3′.

### 4.17 Immunoblots

The cells and mice retinal tissues were lysed in RIPA Lysis containing protease and phosphatase inhibitors (89900, Invitrogen) and followed by ultrasound and centrifugation. The extracted proteins were quantified using a BCA kit (A55860, Invitrogen). The extracts (20–50 μg proteins) were separated on SDS‐PAGE gels and transferred to a PVDF membrane (IPVH00010, Millipore). After blocking with TBST in 5% skim milk, the PVDF membrane was incubated with 1000× diluted antirabbit primary antibodies overnight at 4°C, including RPE65 (Proteintech, 17,939–1‐AP), MnSOD (ab13533, Abcam), GPX4 (#52455, Cell Signaling Technology), FSP1 (#51676, Cell Signaling Technology), 4‐HNE (MA5‐27570, Invitrogen), Hsp70 (ab79852, Abcam), and Tubulin (ab4074, Abcam). After washing thrice with 1× TBST, the membranes were incubated with HRP‐conjugated secondary antibody (1:1000, ab205718, Abcam) at room temperature for 2 h, and the immunoblotting images were captured using an enhanced chemiluminescence detection system (Bio‐Rad). The intensities of the blots were quantified using Quantity One software (Bio‐Rad), and protein expression was normalized to a loading control, Tubulin blot.

### 4.18 GPX4 Ubiquitin‐Degradation

After irradiation, the cells were treated with 10 μM MG132 (HY‐13259, MCE) at 37°C for 4 h to inhibit proteasome‐mediated ubiquitination. The GPX4 protein levels in cell lysates were quantified by immunoblotting. Next, the lysates were immunoprecipitated with 1:100 GPX4 primary antibody (#52455, Cell Signaling Technology) pre‐bound with A/G agarose beads (16–663, Sigma, USA) overnight at 4°C. The eluted fractions were washed with lysis buffer and separated on an SDS‐PAGE gel. The precipitated products were immunoblotted with a ubiquitin antibody (1:1000, #3933, Cell Signaling Technology).

### 4.19 Statistics and Reproducibility

Data are presented as the mean ± standard deviation (SD) from at least three replicates. Significant differences between experimental groups were analyzed using an unpaired Student's *t*‐test. One‐way or two‐way analysis of variance (ANOVA) followed by Dunnett's or Bonferroni's multiple comparison tests was performed using Prism (GraphPad, San Diego, USA). Statistical significance was set at *p* < 0.05.

## Author Contributions

Y.H., Z.Z., and Y.X. conceived and designed the study. Y.H., Z.Z., M.H., X.Z., Q.G., and R.L. performed experiments and data analysis. Y.H., Z.Z., and Y.X. organized results and interpreted data. L.C. and X.L. provided technical support. J.Y., Q.J., and Y.X. supervised the project. Y.H. and Y.X. wrote manuscript drafts and revisions. All authors agree on the manuscript's content and approve the submission.

## Conflicts of Interest

The authors declare no conflicts of interest.

## Supporting information


**Figure S1:** Activities of antioxidant enzymes in dry AMD patients.
**Figure S2:** Activities of antioxidant enzymes in blue light‐irradiated mice with dry AMD phenotype vs. unirradiated mice.
**Figure S3:** Mitochondrial superoxide and hydroxyl radical in MnSOD‐silenced RPE cells exposed to blue light.
**Figure S4:** Quantification of superoxide and hydroxyl radical in MnSOD‐silenced murine primary RPE cells exposed to blue light.
**Figure S5:** Apoptotic cell death induced in MnSOD‐silenced RPE cells.
**Figure S6:** Lipid peroxidation and cell integrity in MnSOD‐silenced RPE cells.
**Figure S7:** Quantification of lipid peroxidation and cell survival in MnSOD‐silenced murine primary RPE cells exposed to blue light.
**Figure S8:** Characterization of mouse Sod2+/− experimental model.
**Figure S9:** The protective effect of MnTBAP on RPE cell survival against ferroptosis.
**Figure S10:** The effect of GPX4 on inhibiting lipid peroxidation in RPE cells.

## Data Availability

Materials and data are included in the article and accessible in the online supplemental information. Additional information required to reanalyze the data presented in this paper is available for reasonable requests to the lead contact, Prof. Yong Xu.
