# Supplementary material for: Superoxide Activates Ferroptosis via the Haber‐Weiss Reaction and Enhances Age‐Related Macular Degeneration
Source: Aging Cell. 2025 Aug 10;24(10):e70195. doi: 10.1111/acel.70195 (PMC12507403; doi:10.1111/acel.70195)
Supplement: Supplementary file 1 — Figure S1: Activities of antioxidant enzymes in dry AMD patients. Figure S2: Activities of antioxidant enzymes in blue light‐irradiated mice with dry AMD phenotype vs. unirradiated mice. Figure S3: Mitochondrial superoxide and hydroxyl radical in MnSOD‐silenced RPE cells exposed to blue light. Figure S4: Quantification of superoxide and hydroxyl radical in MnSOD‐silenced murine primary RPE cells exposed to blue light. Figure S5: Apoptotic cell death induced in MnSOD‐silenced RPE cells. Figure S6: Lipid peroxidation and cell integrity in MnSOD‐silenced RPE cells. Figure S7: Quantification of lipid peroxidation and cell survival in MnSOD‐silenced murine primary RPE cells exposed to blue light. Figure S8: Characterization of mouse Sod2+/− experimental model. Figure S9: The protective effect of MnTBAP on RPE cell survival against ferroptosis. Figure S10: The effect of GPX4 on inhibiting lipid peroxidation in RPE cells. [file ACEL-24-e70195-s001.docx]

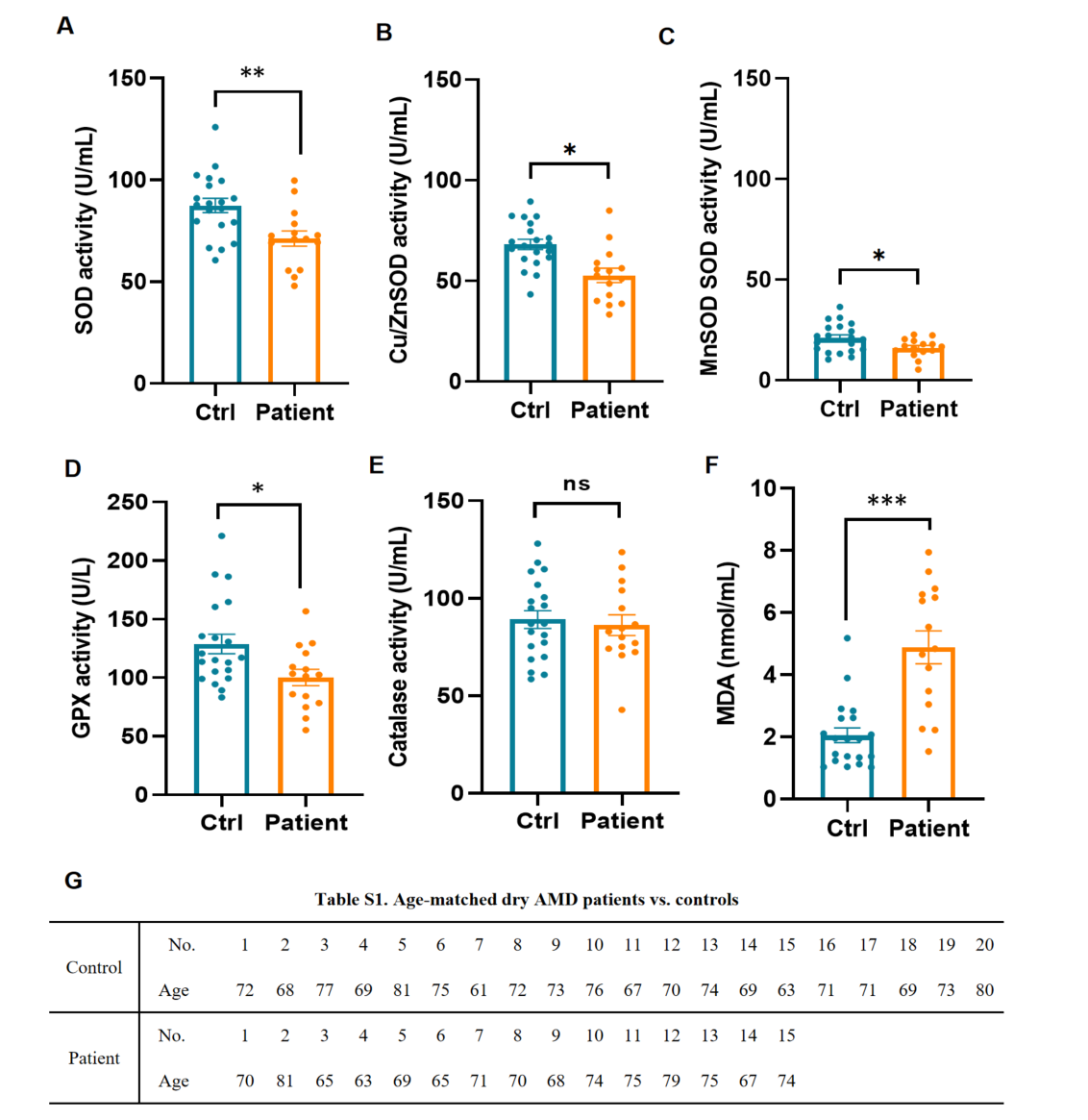


**Figure S1 Activities of antioxidant enzymes in dry AMD patients.**

A-C) The activities of SOD including MnSOD and CuZnSOD in serum samples derived from dry AMD patients (n=15) vs. serum from control donors (n=20). D) The GPX activities in dry AMD patients vs. controls. E) The catalase activities in dry AMD patients vs. controls. F) The levels of MDA in dry AMD patients vs. controls. G) Age-matched dry AMD patients vs. control. The results are presented as the mean ± SD, *p < 0.05, **p < 0.01, ***p < 0.001, ns, no significance (p > 0.05), *t*-test.


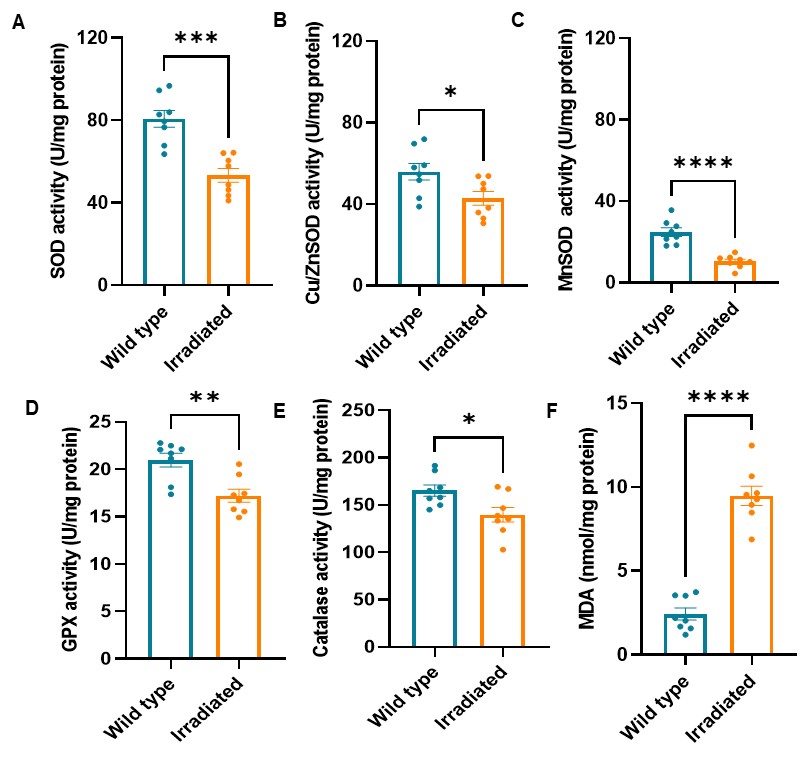


**Figure S2 Activities of antioxidant enzymes in blue** **light-irradiated mice with dry AMD phenotype vs. unirradiated mice.**

A-C) The activities of SOD including MnSOD and CuZnSOD in retina-choroid tissues from light-irradiated mice (n=8) vs. those from unirradiated mice (n=8). D) The GPX activities in samples from light-irradiated mice vs. control mice. E) The catalase activities in samples from light-irradiated mice vs. control mice. F) The levels of MDA in samples from light-irradiated mice vs. control mice. The results are presented as the mean ± SD, *p < 0.05, **p < 0.01, ***p < 0.001, ns, no significance (p > 0.05), *t*-test.


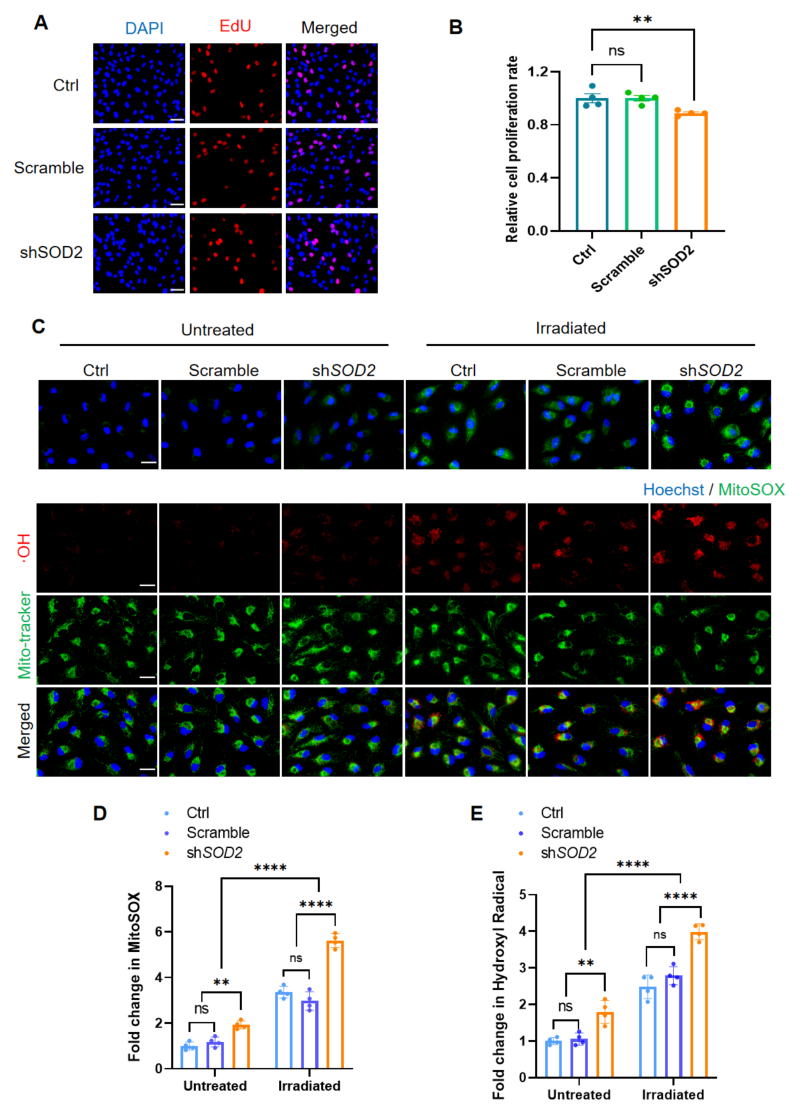


**Figure S3 Mitochondrial superoxide and hydroxyl radical in MnSOD-silenced RPE cells exposed to blue light.**

A,B) Analysis of cell proliferation of MnSOD-silenced RPE cells using an EdU assay. C) Fluorescent image of mitochondrial superoxide and hydroxyl radical in RPE cells exposed to blue light. D, E) Flow cytometry quantifies the levels of mitochondrial superoxide and hydroxyl radicals in the cells (n = 4). The results are presented as the mean ± SD, **p < 0.01, ****p < 0.0001, ns, no significance (p > 0.05), One-way ANOVA. Scale bars: 40 μm (A, C).


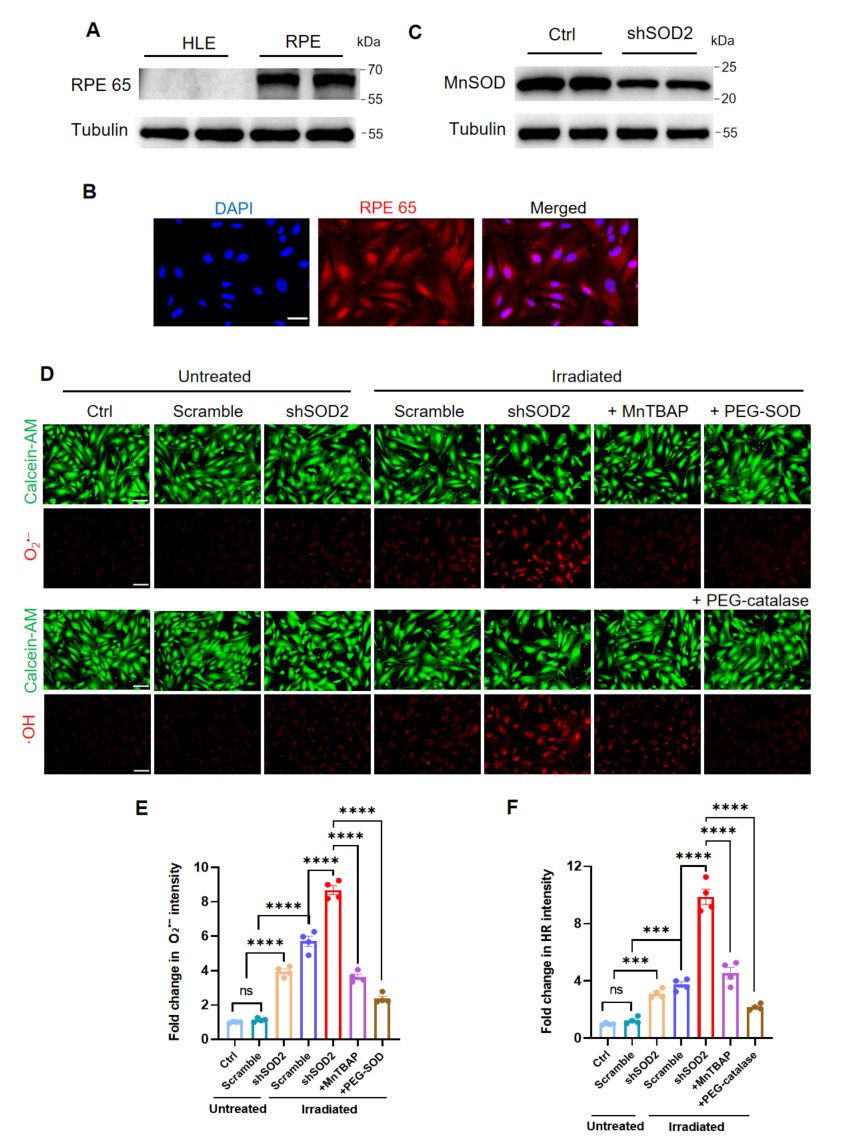


**Figure S4 Quantification of superoxide and hydroxyl radical in MnSOD-silenced murine primary RPE cells exposed to blue light.**

A,B) Characterization of murine primary RPE cells using an RPE65 marker with a lens epithelium control. C) Immunoblots confirm SOD2-silenced in murine primary RPE cells. D) Fluorescent image of superoxide and hydroxyl radical in primary RPE cells exposed to blue light. E,D) Flow cytometry quantifies the levels of cellular superoxide and hydroxyl radicals in the cells (n = 4). The results are presented as the mean ± SD, ***p < 0.001, ****p < 0.0001, ns, no significance (p > 0.05), One-way ANOVA. Scale bars: 40 μm (B,D).


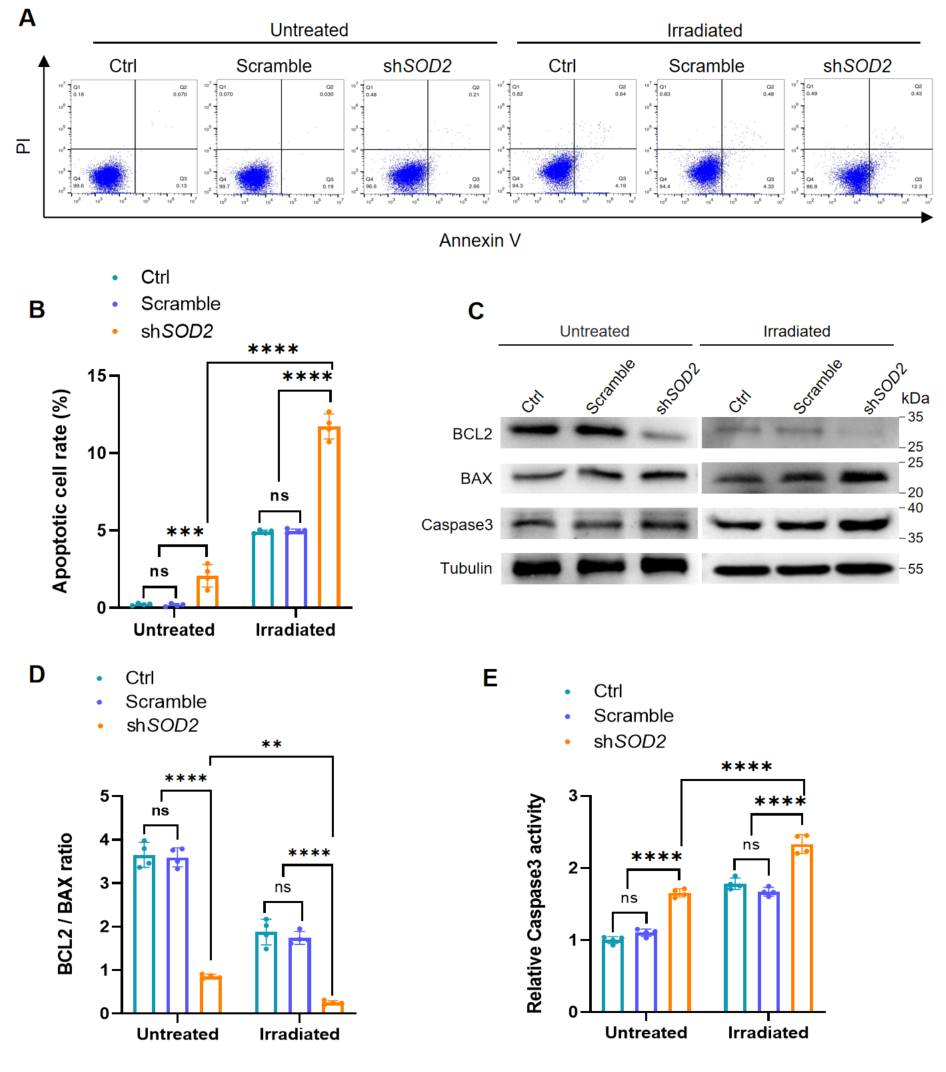


**Figure S5 Apoptotic cell death induced in MnSOD-silenced RPE cells.**

A) Flow cytometry quantifying apoptotic cells after exposure to blue light using propidium iodide (PI) and Annexin-V. apoptotic cells in the top right corners and pre-apoptotic cells in the lower tight corners. B) The apoptotic cell rates in the different groups (n=4). C) Immunoblots of BCL2, BAX, and caspase 3 in the cells. d) The ratio of BCL2 to BAX in the different groups (n=4). E) Caspase 3 activities of the cells (n=4). The results are presented as the mean ± SD. **p < 0.01, ***p < 0.001, ****p < 0.0001, ns, no significance (p > 0.05), two-way ANOVA.


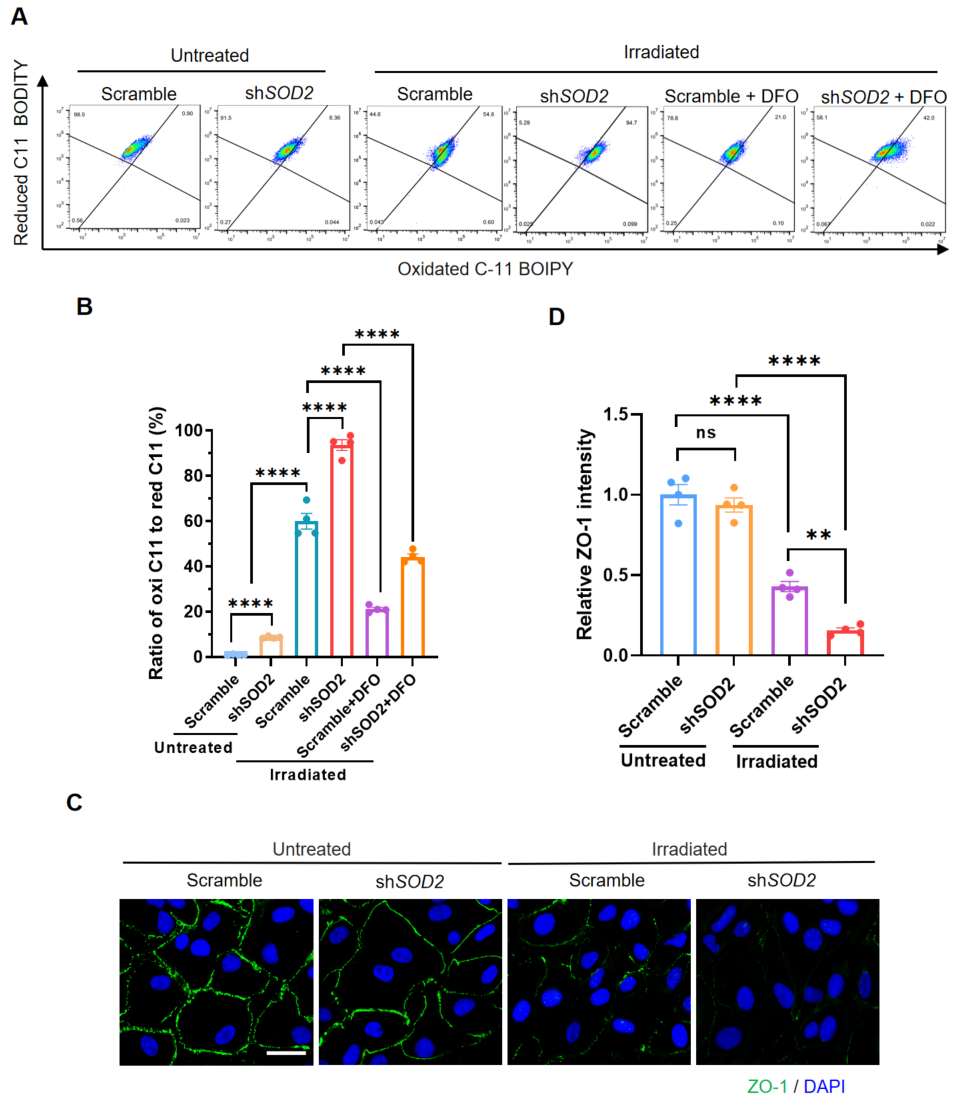


**Figure S6 Lipid peroxidation and cell integrity in MnSOD-silenced RPE cells.**

A) Flow cytometry quantifying oxidized and reduced lipids in RPE cells exposed to blue light. B) The ratio of oxidized lipids to reduced lipids in the cells (n=4). C) Fluorescent image of cell integrity using a ZO-1 fluorescence-conjugated probe with DAPI imaging for nuclei. D) The intensity of ZO-1 staining in the cells (n=4). Scare bar: 20 μm. The results are presented as the mean ± SD. **p < 0.01, ****p < 0.0001, ns, no significance (p > 0.05), two-way ANOVA.


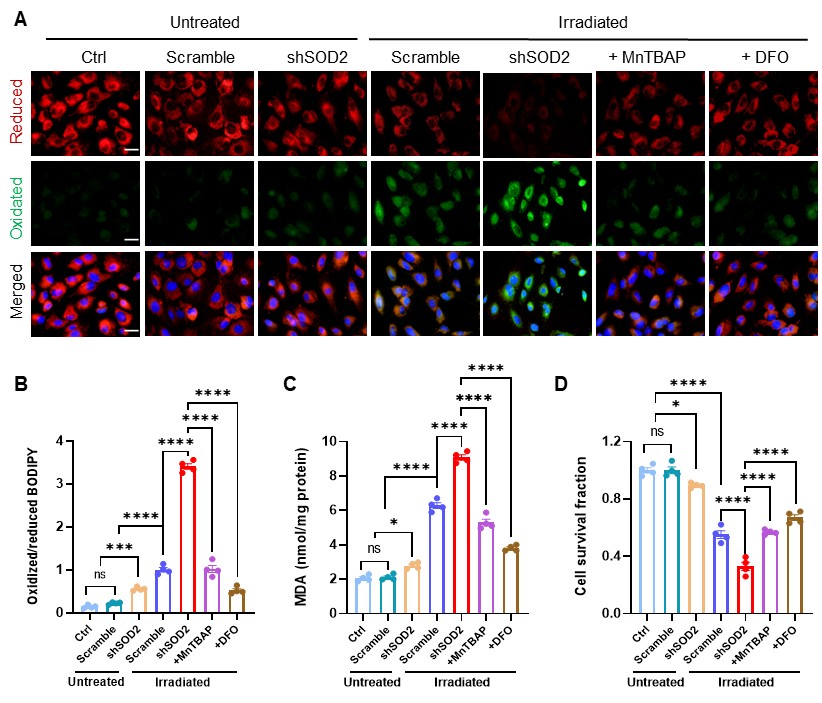


**Figure S7 Quantification of lipid peroxidation and cell survival in MnSOD-silenced murine primary RPE cells exposed to blue light.**

A) Fluorescent image of lipid peroxidation in primary RPE cells exposed to blue light. B) Flow cytometry quantifies the levels of lipid peroxidation in the cells (n = 4), C) Quantification of MDA in the cells (n = 4), D) Cell survival fraction in the cells (n = 4). The results are presented as the mean ± SD, *p < 0.05, ***p < 0.001, ****p < 0.0001, ns, no significance (p > 0.05), One-way ANOVA. Scale bars: 40 μm (A).


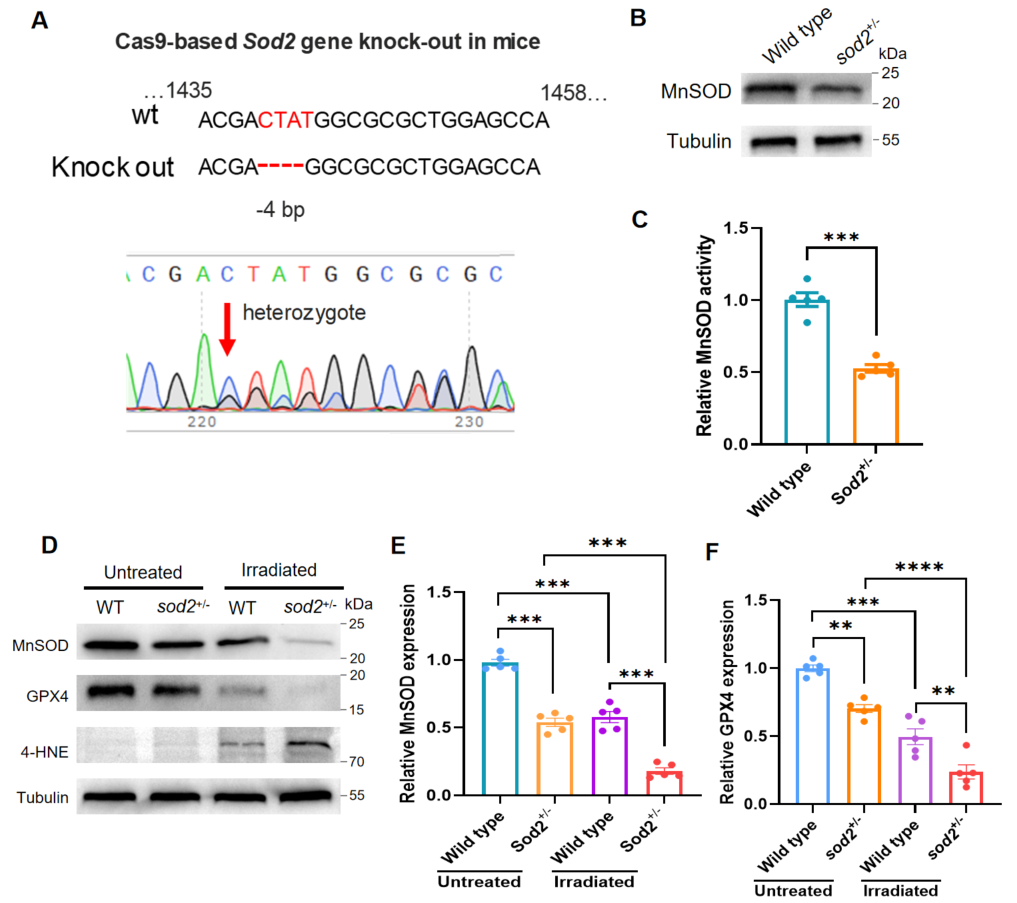


**Figure S8 Characterization of mouse *Sod2*^+/-^ experimental model.**

A) Establishment of a mouse *Sod2*^+/-^ experimental model using a Crispr/Cas9 gene edition approach. Deletion of CTAT four base pairs from the 34th amino acid (aspartate) led to blockage of the MnSOD function by a frameshift amino acid sequence. A red arrow indicates double-sequencing results showing heterozygote mice and frameshift start position. B) MnSOD protein levels in mice retinal tissues. C) MnSOD activities in mice retinal tissues (n=5). D) Immunoblots of MnSOD, GPX4, and 4-HNE expression in *Sod2*^+/-^ mice exposed to blue light. E,F) Relative MnSOD and GPX4 activities in *Sod2*^+/-^ mice exposed to blue light (n=5). The results are presented as the mean ± SD. **p < 0.01, ***, p < 0.001, ****p < 0.0001, *t*-test in (C), two-way ANOVA in (E, F).


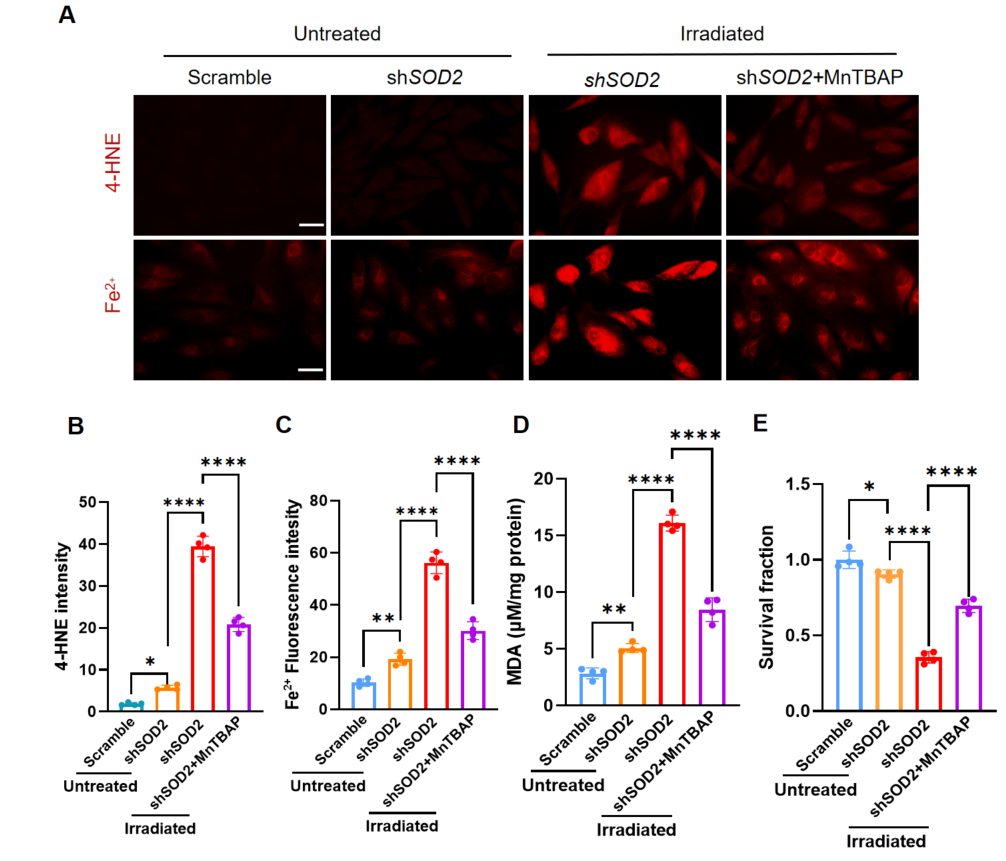


**Figure S9** **The protective effect of MnTBAP on RPE cell survival against ferroptosis.**

A) Fluorescent images of 4-HNE and ferrous ions in MnTBAP-treated MnSOD-silenced cells exposed to blue light. B,C) Flow cytometry quantifying 4-HNE and ferrous ions in the cells (n=4). D) Quantification of MDA in the cells. E) The survival rates of the cells. The results are presented as the mean ± SD. *p < 0.05, **p < 0.01, ****p < 0.0001, one-way ANOVA.


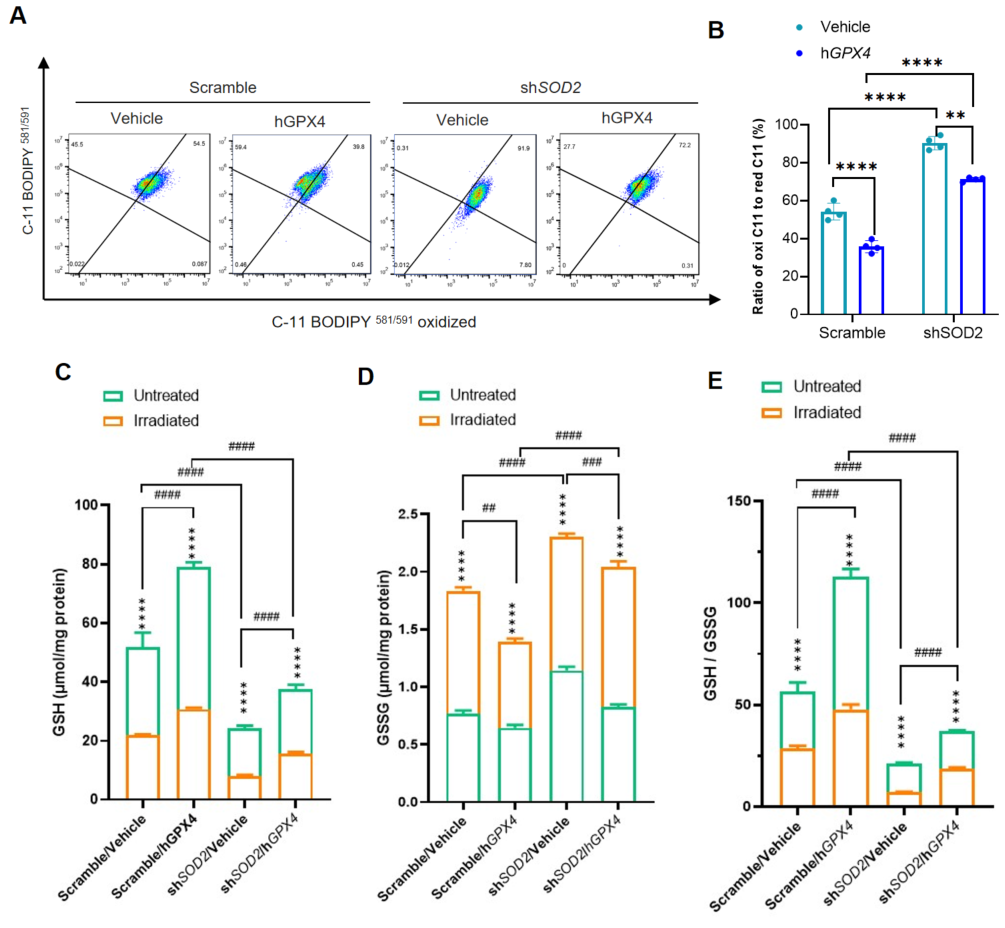


**Figure S10 The effect of GPX4 on inhibiting lipid peroxidation in RPE cells.**

A) Flow cytometry quantifying oxidized and reduced lipids in GPX4-overexpressed RPE cells exposed to blue light. B) The ratio of oxidized lipids to reduced lipids in the cells (n=4). C,D) Quantification of glutathione and glutathione disulfide levels in the cells (n=3). E) The ratio of glutathione to glutathione disulfide in the cells (n=3). The results are presented as the mean ± SD. **p < 0.01, ****p < 0.0001, significant differences in the same groups; ##p<0.01, ###p<0.001, ####p<0.0001, significant differences in the multiple groups, one-way ANOVA in (B), two-way ANOVA in (C-E).
